# The rat osteoarthritis bone score for histological pathology relevant to human bone marrow lesions and pain

**DOI:** 10.1016/j.ocarto.2024.100544

**Published:** 2024-11-27

**Authors:** Daniel F. McWilliams, Mohsen Shahtaheri, Soraya Koushesh, Chitra Joseph, Peter RW. Gowler, Luting Xu, Victoria Chapman, Nidhi Sofat, David A. Walsh

**Affiliations:** a Pain Centre Versus Arthritis and Academic Unit of Injury, Recovery and Inflammation Sciences, University of Nottingham, UK; b NIHR Biomedical Research Centre, Nottingham University Hospitals, UK; c Advanced Pain Discovery Platform, UKRI, London, UK; d Institute for Infection and Immunity, St George's, University of London, London, UK; e Pain Centre Versus Arthritis and School of Life Sciences, University of Nottingham, UK

**Keywords:** Osteoarthritis, Rat, Meniscectomy, Monoiodoacetate, Histology, Bone marrow lesions

## Abstract

**Objectives:**

Histological osteochondral characteristics of inflammation, fibrosis, vascularity, cartilage islands, vessels entering cartilage, thickened trabeculae and cysts are associated with bone marrow lesions (BMLs) in human knee osteoarthritis (OA). We identified and developed a method for scoring comparable pathology in two rat OA knee pain models.

**Methods:**

Rats (n ​= ​8–10 per group) were injected with monoiodoacetate (MIA) or saline, or underwent meniscal transection (MNX) or sham surgery. Pain behaviour (weight bearing asymmetry and mechanical hindpaw withdrawal thresholds (PWTs)) were measured and knee samples obtained. Features associated with BMLs were evaluated using haematoxylin and eosin or Safranin-O stained knee sections. Sections were scored for chondropathy, osteophytes, synovitis and with the human OA Bone Score modified for rats (rOABS). rOABS reliability was assessed with intraclass correlation coefficient (ICC), groups were compared using Mann-Whitney U-tests, and associations examined with Spearman's rho.

**Results:**

OABS features were more prevalent in each OA pain group than in controls. rOABS displayed good inter-rater reliability (ICC ​= ​0.79). rOABS was higher in each model than controls; MIA 3.0 (2.3–4.0) vs vehicle 0.0 (0.0–0.0), and MNX 4.0 (2.3–4.8) vs sham 0.0 (0.0–0.0), each p ​< ​0.003. rOABS was associated with OA cartilage involvement (rho ​= ​0.69, p ​< ​0.001), osteophyte (rho ​= ​0.61, p ​< ​0.001) and synovial inflammation (rho ​= ​0.76, p ​< ​0.001). Higher rOABS was associated with pain behaviour: weight bearing asymmetry (rho ​= ​0.65, p ​< ​0.001) and PWT (rho ​= ​−0.47, p ​= ​0.003).

**Conclusions:**

Subchondral pathology in rat OA models resembles human subchondral BMLs. rOABS reliably measured subchondral pathology and was associated with OA structure and pain behaviour.

## 1 Introduction

Osteoarthritis (OA) is the most prevalent chronic joint disorder. It is characterised by pain, damage to articular cartilage, and osteophyte formation. Subchondral pathology is increasingly recognised as a key aspect of OA. Subchondral bone marrow lesions (BMLs) are visualised on MRI as hypointensity in T1-weighted non fat-suppressed images, and hyperintensity in fluid-sensitive, T2-, proton density-, and intermediate-weighted fat-suppressed and short tau inversion recovery (STIR) images [1]. In human knees BMLs are often adjacent to articular cartilage lesions in the weight-bearing medial tibiofemoral compartment [1]. BMLs are associated with knee pain in people with OA [2].

We previously showed that MRI-identified BMLs in human knee OA are associated with 7 histological pathological characteristics in the affected bone [3]. These characteristics were inflammation, fibrosis, increased vascularity, cartilage islands, vessels entering the cartilage, thickened trabeculae and cysts. We developed and validated the Osteoarthritis Bone Score (OABS) to reflect these histological characteristics that are associated with BMLs in human knee OA [3]. OABS characteristics are linked to dynamic processes, such as inflammation, fibrosis and bone remodelling that might be amenable to therapeutic intervention aiming to reduce pain and structural damage [3]. BMLs were associated with nerve fibres which might directly mediate pain.

Careful back translation of human findings to rodent models might define the nature and consequences of OA pathology, and help evaluate the potential value of new treatments for OA pain and pathology. MRI-defined BMLs have been observed in rodents [4,5], but the small size of rat knees make them challenging to quantify. MRI is unable to fully define pathological mechanisms. We therefore aimed to determine whether histological characteristics of human BMLs are evident in rat models of OA pain, and to adapt OABS for use in rats as an outcome measure suitable for archival and prospectively collected samples.

OA may be induced in rat knees by intra-articular injection of monoiodoacetate (MIA) [6] or by meniscal transection (MNX) [7]. MIA induces chondrocyte cell death and consequent cartilage damage reminiscent of human OA. MNX induces OA pathological change through joint instability and incongruity. Each is associated with pain behaviour in rats. Humans with OA report pain when bearing weight on an OA knee, and weight-bearing asymmetry in rats similarly implies a reluctance to load a painful OA joint. Human OA is associated with evidence of central sensitisation, indicated by increased pain sensitivity at body sites distal to the affected knee [8]. Similarly, reduced withdrawal thresholds to punctate stimulation of the ipsilateral hindpaw in rats with knee OA has been associated with evidence of central sensitisation [9,10].

We here describe subchondral pathology in secondary analyses of rat MIA and MNX knee OA models. This subchondral pathology resembles that previously described as associated with BMLs in human OA knees. We report the development and validation of the rat OABS (rOABS) based on our previously described OABS for human BML-related subchondral pathology.

## 2 Methods

### 2.1 Rat OA models and pain behaviour

Male Sprague Dawley rats (weighing 160–190 ​g) were purchased from Charles River UK. Rats were acclimatized (3–7 days) and habituated to handling by the experimenter before any experimental procedures started. Studies had the approval of the University of Nottingham's Animal Welfare and Ethical Review Board and were conducted in accordance with the requirements of the UK Home Office Animals Scientific Procedures Act (1986). Data reported here represent secondary analysis of samples and pain behaviour data from rats previously reported in a studies of OA pain models [11,12]. Outcomes of cartilage and osteophyte scores and pain behaviour for these animals have previously been reported. All histological scores reported herein were determined each in a single assessment using sections in random order pooled from the original studies, by assessors who were blinded to previously reported scores, experimental group, and pain behaviour data. We have previously shown that MIA and MNX models display OA pathology >7 days prior to the time of euthanasia used in the current experiments [11,12]. All outcome measurements were carried out by an experimenter blinded to randomized treatments, except that pain behaviour assessments were undertaken in vivo within separate MNX and MIA studies.

The MIA model of OA pain was induced with intraarticular injection of 1 ​mg MIA in 50 ​μl saline (n ​= ​10) in the knee, and controls (n ​= ​10) underwent saline injection under brief anaesthetisa (isoflurane 3 ​% induction, 1 ​L/min O_2_). Rats underwent assessments of weight-bearing asymmetry (percentage) and hind paw withdrawal thresholds to von Frey filaments (g) throughout the experiment using established methods [12]. Pain behaviour data for the current study comprised the median of the 3 time points (days 21, 24 and 28 after model induction) prior to euthanasia on day 28.

For the MNX model of OA pain, rats were anaesthetised with isoflurane (3 ​% induction, 2–2.5 ​% maintenance; 1 ​L/min O2), and local anaesthetic EMLA cream was applied to the left hind limb. The MNX model was induced by surgical full thickness transection of the meniscus (n ​= ​10), controls were subjected to a sham operation that preserved meniscal integrity (n ​= ​8) [12]. Pain behaviours were assessed throughout the experiment using the same established methods as used for the MIA model [11]. Data used herein comprised the median of the last 3 time points (days 35, 38 and 42 after model induction) prior to euthanasia on day 42.

After euthanasia, knee samples were dissected to expose the internal structures of the knee joint and fixed in formalin and embedded in paraffin wax for sectioning. Coronal sections (5 ​μm) of each knee were analysed from 3 levels (depths) from anterior to posterior, each level separated by approximately 200 ​μm. Replicate sections from each level were stained using haematoxylin and eosin (H&E), or safranin-O, fast green and haematoxylin.

The protocol for this study was not pre-registered.

### 2.2 Identification and scoring of OABS features

**Fig. 1:**
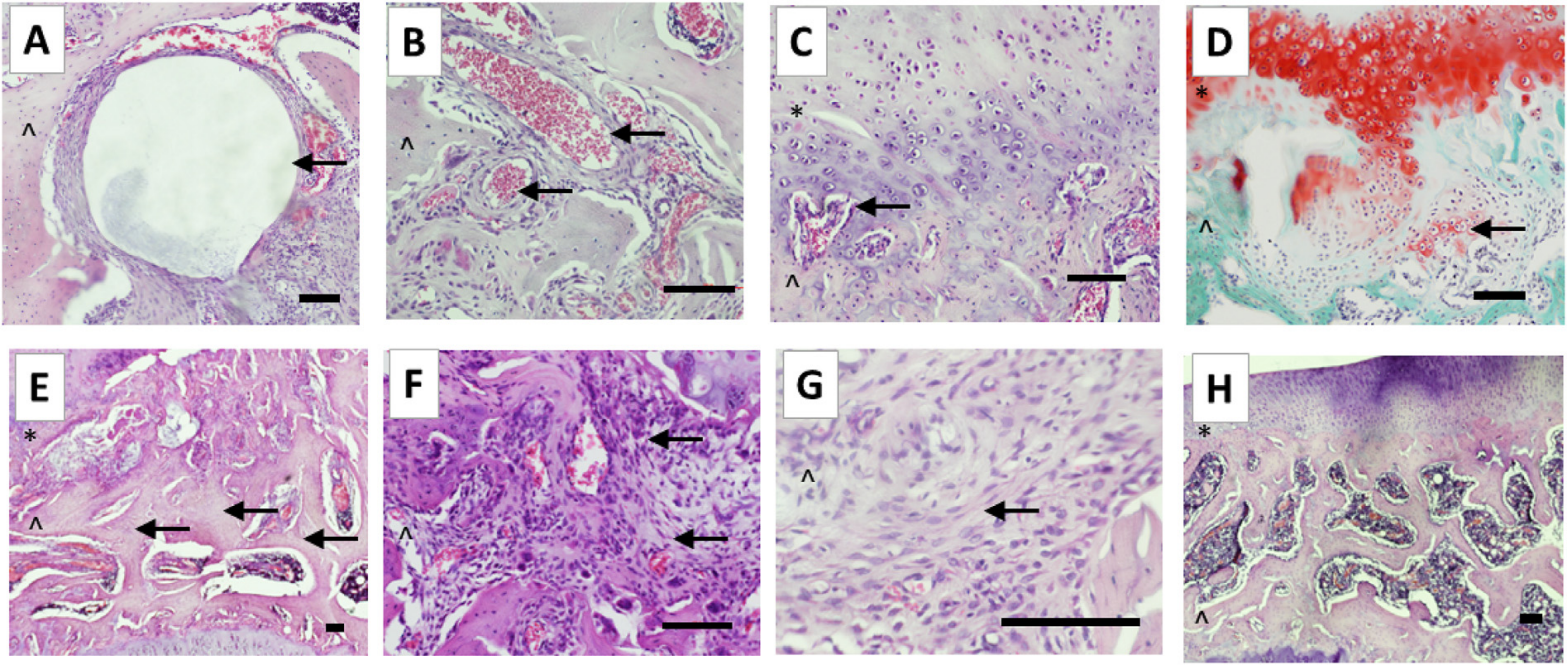
Features of the OABS in rat medial tibial plateau. A: Cyst (indicated by arrow). B: Vessels (arrows indicate vessels within the subchondral region). C: Vessels invading the cartilage (vessel within the cartilage indicated by arrow), D: Cartilage island (indicated by arrow). E: Thickened trabeculae (trabeculae indicated by arrows). F: Inflammation (examples of inflammatory cell infiltrates indicated by arrows). G: Fibrosis (example indicated by arrow). H: Normal appearance of subchondral region (at low power), with no cysts, no regions of inflammation, no regions of fibrosis, no cartilage invasion, no cartilage islands. ∗ ​= ​articular cartilage, ˆ ​= ​subchondral region.scale bars ​= ​100 ​μm.

The subchondral region of interest was selected to be the tissues between the growth plate (which was not assessed) and the articular cartilage, and was restricted, as in the human OABS, to the medial tibial plateau (see Supplement 1 and Fig. 1). Each feature of human OABS [3] was sought in the subchondral region of medial tibial plateaux. Human OABS scoring criteria were modified for the new rOABS where necessitated by the smaller dimensions and specific characteristics of normal rat compared to human knees. Characteristics were scored using paired H&E and Safranin O stained sections, as either present [1] or absent (0) and a summated score from 0 to 7 was generated for each of the 3 coronal levels. H&E staining was optimal for rOABS scoring for all criteria except for cartilage islands and vessels entering the non-calcified cartilage, when Safranin O staining was optimal. rOABS scoring was performed with the group and code identity concealed, and therefore blinded to previously reported scores, experimental group, and pain behaviour data.

### 2.3 Osteoarthritis scoring

Scoring for OA features of cartilage loss/damage, osteophyte formation and synovial inflammation was performed as previously published [7]. Cartilage score assessed the depth of any lesion within the cartilage, ranging from superficial (=1) to a lesion reaching the underlying bone (=5). Involvement score assessed whether a lesion covered 0/3, 1/3, 2/3 or 3/3 of the cartilage surface. Cartilage Involvement score was the product of the Cartilage and Involvement scores. Osteophytes were scored from 0 to 3 with increasing size. Synovial inflammation score ranged from 0 to 3 with increasing cellularity and thickness of the lining. Scoring was by one assessor (MS) using the H&E-stained sections. All scoring was performed with the group and code identity concealed, and therefore blinded to previously reported scores, experimental group, and pain behaviour data.

### 2.4 Statistical analysis

Data are presented as median (IQR) or mean (95 ​% Confidence Interval). Optimal number of sections was determined by comparing the coefficient of variation between the blinded scoring of sections from 1, 2 or 3 levels, for all knees and confirmed in the subgroup with rOABS>1, to reduce the influence of 0 scores in the controls. Decisions were reached based upon the perceived improvements in precision compared to decreases in efficiency from analysing greater numbers. Reliability of rOABS was assessed between observers scoring the same sections using twoway mixed effects intraclass correlation coefficients for a single measurement with absolute agreement (ICC(2,1), 95 ​% CI) and intraobserver reliability by the same observer scoring the same sections on different occasions (ICC(3,1), 95 ​% CI). Reliability was assessed between 2 experienced human OABS scorers who had no prior consultation or specific training for rat OABS and derived scoring rules from the rOABS atlas (supplement 1). Reliability was also assessed between the rOABS developer (SS) and a newly trained scorer, to evaluate the written protocol given in Supplement 1. rOABS scores were compared between OA models and their respective controls (MIA vs vehicle; MNX vs sham surgery) using Mann-Whitney U-tests due to small group sizes. Unpaired t-tests were used to generate approximations of the mean (95 ​% CI) difference between groups. Associations of rOABS with pain behaviour (weight-bearing asymmetry or paw withdraw threshold) used Spearman's rho. A post-hoc power calculation for correlation coefficients of all samples (n ​= ​38) showed 91 ​% achieved power when r ​= ​0.50. When OA models were examined alone, a post-hoc power calculation for correlation coefficients when n ​= ​20 showed 64 ​% achieved power for the same r ​= ​0.50, but 13 ​% achieved power when r ​= ​0.20 (G∗power software, Kiel University, Germany). Linear regression analyses were used to assess possible effects of model/study (MNX vs MIA) on pain behaviours and their associations with rOABS. Binary scores were compared between groups by χ^2^ test.

## 3 Results

### 3.1 Rat OA pain models, pathology and pain behaviours

The MIA and MNX model each induced OA-like pain behaviour compared to their respective control groups (Table 1).

**Table 1:** Pain behaviour in OA models compared to non-arthritic controls.

	MIA	Vehicle	p	MNX	Sham	p
**Osteoarthritis features**						
Cartilage involvement score	3 (1–8)	0 (0–0)	0.012	4 (1–8)	0 (0–0)	0.009
Cartilage score	2 (1–4)	0 (0–0)	0.012	3 (1–5)	0 (0–0)	0.001
Involvement score	2 (0–2)	0 (0–0)	0.012	1 (0–2)	0 (0–0)	0.001
Osteophyte score	0 (0–0)	0 (0–0)	>0.999	1 (0–3)	0 (0–0)	0.008
Synovial inflammation score	3 (1–3)	0 (0–0)	0.006	3 (1–3)	0 (0–0)	0.006
**Pain characteristics and measurements**						
Weight bearing asymmetry prior to OA induction (%)	−0.7 (−2.8 to 0.9)	−2.1 (−3.8 to 3.7)	0.320	−0.4 (−2.4 to 2.4)	2.0 (0.7–4.0)	0.360
Weight bearing asymmetry during last 7 days prior to termination (%)	19.5 ​% (12.6 ​%–23.5 ​%)	−0.3 ​% (−2.3 ​%–0.5 ​%)	<0.001	5.6 ​% (0.8 ​%–9.6 ​%)	0.9 ​% (−0.7 ​%–1.0 ​%)	0.040
Paw withdrawal threshold prior to OA induction	15 (13–26)	15 (10–26)	0.791	26 (18–26)	21 (14–26)	0.203
Paw withdrawal threshold during last 7 days prior to termination (g)	4 (3–8)	15 (15–26)	0.002	10 (7–17)	14 (10–16)	0.622

Cartilage score assesses the depth of any lesion within the cartilage, ranging from superficial (=1) to a lesion reaching the underlying bone (=5). Involvement score assesses whether a lesion covers 0/3, 1/3, 2/3 or 3/3 of the cartilage surface. Cartilage Involvement score is the product of the Cartilage and Involvement scores. Osteophyte score ranged from 0 to 3 with increasing size. Synovial inflammation score ranges from 0 to 3 with increasing cellularity and thickness of the lining. Weight bearing asymmetry was defined as the difference in the amount of weight between the right contralateral control limb and the left ipsilateral treated limb divided by the sum of the weight right and left limbs ​× ​100. All data are median (IQR). Mean (95 ​% CI) differences are presented in Supplement 2.

Analysis of the OA model knee joints collected at the study endpoint showed significant histological changes in both models consistent with OA (Table 1), with negligible changes observed in sham or vehicle controls. When MNX and MIA models were compared to each other for OA scores at study endpoint, the chondropathy (Cartilage-Involvement) score (median (IQR) MNX 4 (1–8); MIA 3 (1–8), p ​= ​0.939), and the subscores for cartilage lesion depth (MNS 3 (1–5); MIA 2 (1–4), p ​= ​0.558) and involvement, defined by cartilage lesion width, (MNX 1 (0–2); MIA 2 (1–2), p ​= ​0.814) were similar between the two models. Synovial inflammation scores also were similar between both models (3 (1–3) for both MNX and MIA, p ​= ​0.573). The MIA model had negligible osteophytes, with osteophyte scores that were similar to vehicle controls, and were less than in the MNX model (MNX 1 (0–3); MIA 0 (0–0), p ​= ​0.002). Estimates of the mean (95 ​% CI) differences between groups are presented in Supplement 2.

### 3.2 Subchondral pathology of the rat osteoarthritis bone score

All 7 features of the human OABS [3] were identified in the subchondral regions of the medial tibial plateaux of the MNX/MIA model knees (see Fig. 1 and for details regarding scoring, see Supplement 1). Cysts, cartilage islands, regions of fibrosis, regions of inflammation, increased vascular density, vascular growth into the articular cartilage and thickened trabeculae were each observed in at least 1 sample (ranging from 10 ​% to 80 ​% prevalence in each OA model). Prevalence of each individual feature scored by rOABS differed by no more than 20 ​% between MNX and MIA, except that the prevalence of trabecular thickening was 70 ​% in the MIA and 30 ​% in the MNX model (Table 2, p ​= ​0.175).

**Table 2:** Prevalence of OABS features in rat knee OA models.

	MNX	Sham operation	MIA	Vehicle
Cyst	20 ​%	0 ​%	10 ​%	0 ​%
Fibrosis	70 ​%	0 ​%	80 ​%	0 ​%
Vasculature	30 ​%	0 ​%	30 ​%	0 ​%
Inflammation	70 ​%	0 ​%	80 ​%	0 ​%
Cartilage island	80 ​%	0 ​%	60 ​%	0 ​%
Vessels into cartilage	20 ​%	0 ​%	20 ​%	0 ​%
Trabeculae	70 ​%	0 ​%	30 ​%	0 ​%

Values give percent of medial tibial plateaux from each study group displaying each OABS feature in sections from any of the 3 coronal levels that were assessed.

A subchondral tissue region of interest was defined for scoring the features of OABS. The depth of this region encompassed the subchondral bone of the medial tibial plateau from the articular cartilage to the growth plate. The width comprised all of the medial tibial plateau except for the regions at the immediate medial edge and the central region where ligaments influenced the histology of subchondral bone. Three necessary modifications from human OABS scoring criteria [3] were made to incorporate differences between species [1]: all channels entering articular cartilage were counted due to absence of visible tidemarks in experimental rats [2], criteria for trabecular thickening reflected the small dimensions of rat knees [3], 15 or more vessels within the subchondral region of interest was selected as the threshold for high vascularity. Blood sinusoids within subchondral bone were excluded from vessel counts. Rules for scoring each aspect as no (0) or yes [1] were derived to give a final scoring system from 0 to 7 similar to the human OABS (Supplement 1).

Similar rOABS mean (1.6, 1.6 and 1.5), sd (2.0, 2.0 and 2.0), and coefficients of variation (1.25, 1.25 and 1.33) were derived for 1, 2 and 3 replicate sections from different levels of rat medial tibial plateaux. When cases with rOABS≥1 were selected (to remove any influence of zero values), coefficients of variation for 1, 2 and 3 sections were 0.29, 0.31 and 0.33 respectively. As increasing numbers of replicate sections did not importantly improve coefficient of variation, a single section per case from the first level of the block was used for subsequent calculation of rOABS.

Reliability of repeat measurements of rOABS assessed through comparisons of 2 experienced human OABS assessors (MS, SK) yielded an ICC(2,1) 0.64, (95 ​% CI, 0.27 to 0.83), p ​= ​0.001. Comparisons of a newly-trained assessor (CJ) with an experienced assessor (MS) yielded an ICC(2,1) ​= ​0.79 (95 ​% CI, 0.63 to 0.88), p ​< ​0.001. Intra-observer reliability for an experienced observer (MS) scoring on 2 separate occasions was ICC(3,1) ​= ​0.88 (0.77–0.94), p ​< ​0.001.

### 3.3 rOABS association with OA pathology and pain behaviour

**Fig. 2:**
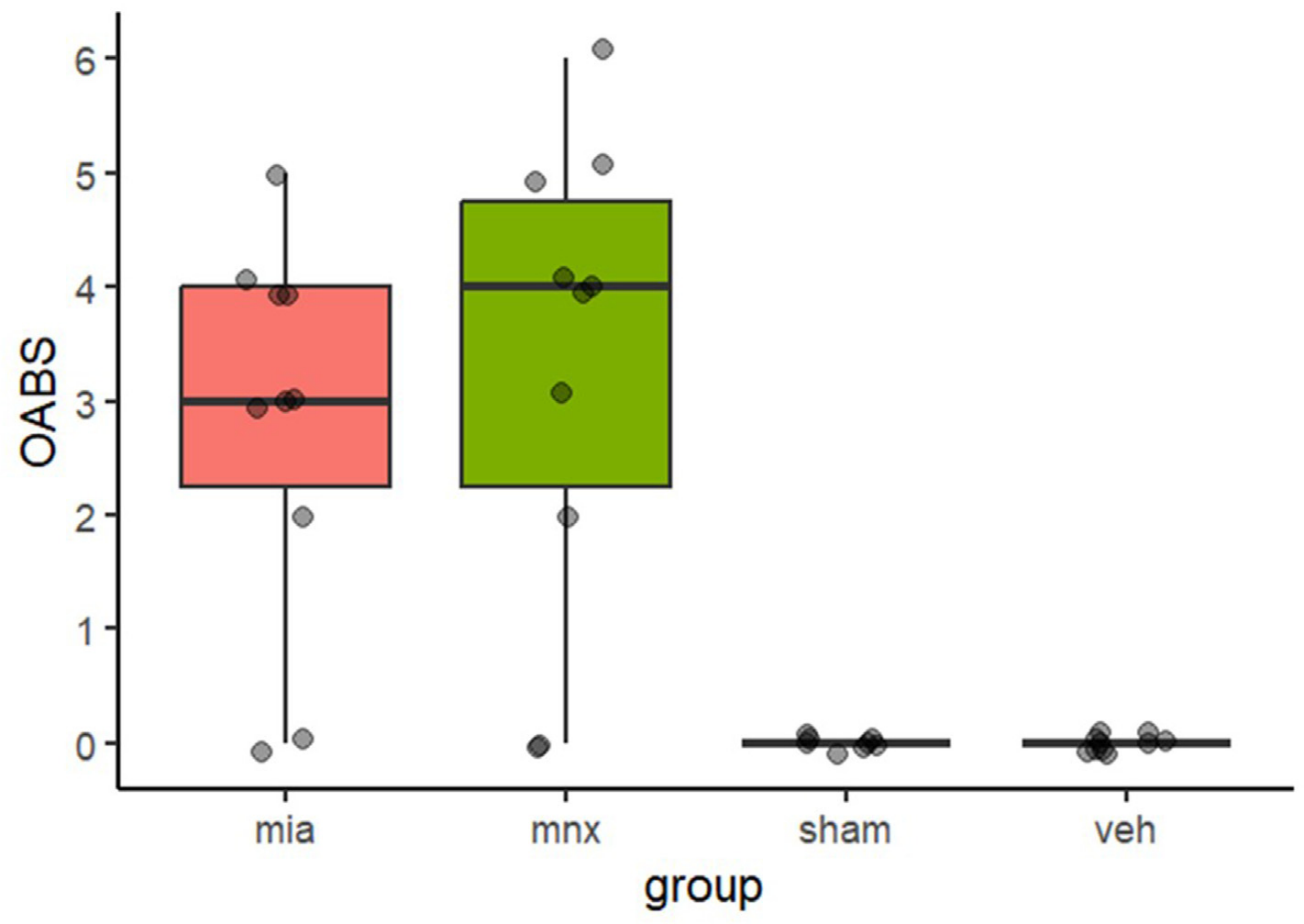
Box and whiskers plot of rOABS median, IQR and range, with each datapoint included and jittered. Key, mia ​= ​monoiodoacetate, mnx ​= ​meniscal transection, sham ​= ​sham surgery, veh ​= ​vehicle.

rOABS scores were higher in each model than their controls (median (IQR)); MIA 3.0 (2.3–4.0) vs vehicle 0.0 (0.0–0.0), p ​< ​0.001, and MNX 4.0 (2.3–4.8) vs sham 0.0 (0.0–0.0), p ​= ​0.002. There was no significant difference between rOABS for MIA vs MNX (p ​= ​0.439), nor between vehicle vs sham operated controls (p ​> ​0.99) (see ​Fig. 2).

**Fig. 3:**
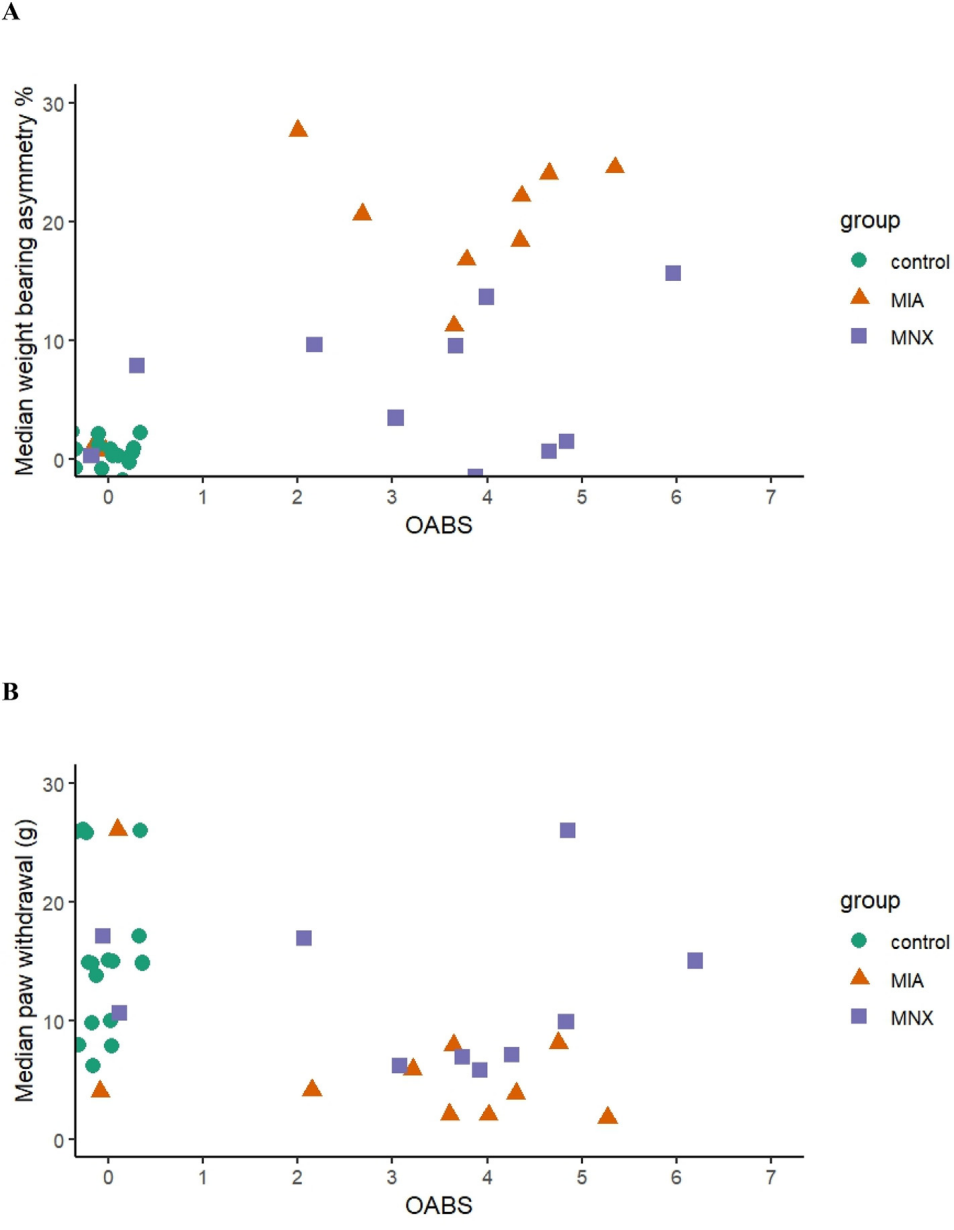
A: rOABS vs weight bearing asymmetry (percentage) and B: paw withdraw threshold (g). Points are jittered to prevent overlapping.

The histological OA severity scores showed significant correlations (rho (95 ​% CI)) with rOABS for cartilage involvement (0.69 (0.47–0.83), p ​< ​0.001), osteophyte (0.61 (0.36–0.78), p ​< ​0.001) and synovial inflammation (0.76 (0.57–0.87), p ​< ​0.001). OA features across each group are shown in Table 1. Higher rOABS was associated with more severe pain behaviour, as indicated by greater weight bearing asymmetry percentage (0.65 (0.42–0.80), p ​< ​0.001) and lower paw withdrawal threshold (g) (−0.47 (−0.69 to −0.18), p ​= ​0.003) (Fig. 3). Linear regression analysis indicated that weightbearing asymmetry was less in the MNX than the MIA study (beta coefficient (95 ​% CI): −5.37 (−9.56 to −1.18), p ​= ​0.014), but PWT did not differ significantly between models/studies (beta coefficient (95 ​% CI): 0.96 (−3.81 to 5.73), p ​= ​0.686)). Associations between rOABS and pain behaviour remained significant after adjusting for model/study (beta coefficients (95 ​% CI) for weight bearing asymmetry: 2.97 (1.97–3.97), p ​< ​0.001, and for paw withdraw threshold: −1.62 (−2.76 to −0.48), p ​= ​0.007). Subgroup analyses of rats after MNX, or after MIA injection, were not sufficiently powered to confirm significant association between rOABS and pain behaviours (weight-bearing asymmetry rho (95 ​% CI) ​= ​0.15 (−0.31 to 0.56), p ​= ​0.505; von Frey hair rho (95 ​% CI) ​= ​−0.14 (−0.55 to 0.32), p ​= ​0.544).

## 4 Discussion

We show that rat models of knee OA induced by intra-articular injection of MIA, or by meniscal transection (MNX), each display similar subchondral pathology to that previously described in human OA BMLs. Furthermore, subchondral pathology in these rat OA pain models is associated with cartilage damage, osteophytosis and synovitis, and with pain behaviour. We have developed and validated a histopathological scoring system for subchondral OA pathology in rat knees (rOABS). Subchondral pathology is an integral aspect of rat OA.

We found that rat OA knee pain models induced by either chemical or surgical insult, each displayed subchondral pathology resembling that in human OA BMLs, as well as displaying chondropathy and osteophytosis. This suggests that subchondral pathology is a key aspect of OA in general. OA is a disease of the whole joint, including subchondral bone. Future studies should consider the subchondral pathology of OA models, and account for this as well as osteophytes, cartilage loss and inflammation.

Subchondral pathology in each rat model studied here was associated both with weight-bearing asymmetry and with reduced hindpaw withdraw thresholds to punctate stimulation. Similarly BMLs in human OA have been associated with pain, and, in particular, weight-bearing pain [2]. Pain behaviour in rats is an indirect assessment of pain or sensitivity, and may be influenced by multiple factors in addition to knee OA. We minimised this by using standardised procedures and experimental environment, and addressed measurement error by combining pain behaviour data collected during the last 7 days prior to euthanasia, time points that we have previously shown that the models display stable OA pathological change [11,12].

We have developed and validated a scoring system (rOABS) for histological changes in rat subchondral bone that are associated with OA structural severity, and with pain behaviour. Instructions with an illustrative atlas for rOABS are presented in supplement 1. rOABS back-translates human OABS, enabling experimental investigation in rats to be placed within the context of human subchondral pathology. All of the features we previously associated with BMLs in human knees were also found in the subchondral bone of rat OA pain models. rOABS could distinguish between pathologic and control samples with relatively modest sample sizes of 8–10. rOABS displayed inter-observer reliability, indicating that it can be applied by different researchers, enabling comparison between studies and models, prospectively and retrospectively. Use of a comprehensive scoring guide/atlas (see Supplement 1) aimed to promote reliable scoring, even with no discussion or training between 2 observers. However, unsurprisingly it was possible to improve inter-observer reliability through discussion and pre-training on a separate set of slides prior to scoring of the samples.

rOABS displayed validity both in MIA and in MNX models of rat knee OA. These two distinct models, with different aetiologies, each displayed the histological features captured by the scoring system, whereas controls did not. rOABS was closely aligned to the human OABS, with only minor modification due to the smaller size and relative immaturity of experimental rats. rOABS, might therefore be widely applicable, although use in other models and species requires further validation. rOABS has potential as an outcome measure for experimental studies aiming to reduce OA symptoms and structural damage by targeting subchondral pathology.

Subchondral bone and BMLs are likely drivers of pain in human OA [1]. OA is a disease that affects multiple joint tissues. Cartilage and synovium, and additional central mechanisms, may also influence OA pain [8,13]. Subchondral bone, both in humans and in rats, has a rich innervation by peptidergic sensory fibres [14], and human OA BMLs also contain sensory nerves [3]. Breaching of the osteochondral junction is associated with growth of sensory nerves into OA cartilage [14,15], and angiogenesis, new blood vessel growth, is associated with nerve growth [14,15]. Disruption of the osteochondral junction might also allow mediators from the synovial fluid to stimulate or sensitise nociceptors in the subchondral bone. BML-like pathology in human subchondral bone is associated with increased osteoclast numbers [16], which may not only mediate altered trabecular morphology, but also release nerve growth factor and generate an acidic microenvironment which may sensitise subchondral nociceptors. Characteristics addressed by rOABS might therefore directly or indirectly contribute to OA pain through the activation, sensitisation or growth of subchondral and osteochondral sensory nerves.

Our research has some strengths, but is also subject to several limitations. Subchondral bone is unlikely to be the only source of OA pain. Human knees with OA may be painful even in the absence of BMLs, and some of the experimental rats in the current study displayed rOABS scores of zero. Cartilage, bone and synovial OA pathology are closely associated with each other, and further research is required to determine direct pain mechanisms, rather than pain associations that can be explained by concurrent pathology in other joint tissues. rOABS was associated with OA structural severity, and we were unable to include within-joint controls due to the small size of rat knees, unlike in the development of the human OABS [3]. Application of rOABS by different research teams without training displayed statistically significant reliability, but the confidence intervals of the ICC did not exclude poor levels of reliability. Direct comparisons between different studies must be made with caution. However, training of a new assessor within the same team provided highly reliable findings, as did re-scoring by the same observer. Our findings indicate a plausible but not conclusively direct, causal association between rOABS and pain. rOABS evaluates subchondral pathology associated in human knees with MRI-defined BMLs, but is not a direct measure of BMLs. BMLs can represent heterogeneous pathology and are not restricted to OA joints. Additional measurements of other parameters might improve our understanding of the context of rOABS in OA models. Interventional research is required to determine whether histopathological characteristics addressed by rOABS are those that explain previously observed associations between BMLs and pain or structural damage. Research in larger animals and humans is needed to explore spatial relationships between subchondral pathology, cartilage damage and biomechanical forces. However, histological evaluation using rOABS avoids some of the technical and ethical difficulties of using MRI in rats. We restricted our analyses to the medial tibial plateau, which is a weight-bearing region most commonly affected in human OA, and reflects a site of maximal OA pathology after medial meniscal transection. Further research would need to confirm whether the changes we observed are representative of other joints or knee compartments. Joint tissues and pain behaviour data used in the current report were derived from 2 studies respectively investigating MIA or MNX models of OA pain. Reuse of tissues and data supports ethical reduction in animal experiments. However, comparisons between models may be confounded by other contextual factors that can differ between studies. Despite this, we did not find important effects of model/study on the observed associations between rOABS and pain behaviour. Animal models are developed to encourage homogeneity between animals and maximise power to detect effects of interventions, whereas heterogeneity is necessary to demonstrate associations. Exclusion of controls and separate analysis of single models reduces this heterogeneity and therefore reduces power to detect associations. However, inclusion of different models and control animals within a single analysis raises the potential for unmeasured confounding factors, rather than subchondral pathology, to explain our observed associations with pain behaviour. As with all semiquantitative scoring methods, rOABS has a subjective component and is subject to measurement error. The good reliability demonstrated in this study, ability to distinguish between OA and control rat groups each of only 8 animals, and significant associations with pain behaviour and other indices of OA pathology, indicate that this subjectivity and measurement error do not preclude rOABS utility. However, it remains important to ensure appropriate blinding of scorers to experimental details, and to confirm reliability of new scorers. rOABS appears generalisable, but requires validation in other species, models and joints. rOABS is designed to evaluate subchondral pathology in rat OA pain models and is not intended to be a holistic measure of OA structural severity. Although significantly associated with scores for chondropathy, osteophytes and synovitis, none of these alone adequately measures the totality of OA pathology. rOABS therefore should be used to complement rather than replace assessment of other joint compartments, for example using methods recommended by OARSI for histological assessment of rat OA [17].

In conclusion, subchondral pathology in rat OA pain models induced by MIA or MNX resembles human BMLs, and is associated with OA pathology in cartilage, osteophytes and synovitis. rOABS reliably measures rat OA subchondral pathology, and is associated with pain behaviour. Use of rOABS in rat OA models should help identify novel interventions targeting subchondral bone to improve pain and structural damage.

## Data availability

Please contact corresponding author.

## CRediT author contribution

Conceptualization; DM, DW, NS, SK, MS.

Data curation; DM, MS, CJ.

Formal analysis; DM.

Investigation; DM, DW, NS, SK, MS, CJ, VC, PG, LX.

Methodology; DM, DW, NS, SK, MS, CJ, VC, PG, LX.

Project administration; DW, NS, DM, VC.

Supervision; DM, DW, NS, VC.

Validation; DM, MS, CJ, SK.

Roles/Writing – original draft; DM, DW, NS, SK, MS, CJ, VC, PG, LX.

Writing – review & editing. DM, DW, NS, SK, MS, CJ, VC, PG, LX.

## Funding

Supported by the Rosetrees’ Trust (Grant number M11-F3), the 10.13039/100010269Wellcome Trust Institutional Support Fund (ISSF) (Grant number 204809/16/Z). The views expressed are those of the author(s) and not necessarily those of the funders.

## Declaration of competing interest

DAW has grant support from 10.13039/100004312Eli Lilly & Company, 10.13039/100009032Pfizer Ltd, GSK Ltd, 10.13039/501100024580Orion Pharma, 10.13039/100011110UCB.

DAW - Consultancy fees paid to University of Nottingham; Contura International A/S, Glaxo SmithKline, AKL Research and Development Ltd, Pfizer Ltd, Abbvie Ltd, Ely Lilly & Co.Ltd, Galapagos Ltd., Reckitt Benckiser Health Ltd.

DAW - Speaker fees paid to University of Nottingham; Medscape International, Pfizer Ltd.

DFM has grant support from 10.13039/100004312Eli Lilly & Company, 10.13039/100009032Pfizer Ltd.

NS has grant support from 10.13039/100008021Bristol Myers Squibb, 10.13039/100004312Eli Lilly & Company and 10.13039/100009032Pfizer Ltd.
