# Supplementary material for: The rat osteoarthritis bone score for histological pathology relevant to human bone marrow lesions and pain
Source: Osteoarthr Cartil Open. 2024 Nov 27;7(1):100544. doi: 10.1016/j.ocarto.2024.100544 (PMC11665527; doi:10.1016/j.ocarto.2024.100544)

OSTEOARTHRITIS BONE SCORE (OABS) FOR RAT KNEE OA MODELS (medial tibial plateau)

The rat knee joint, stained with Safranin-O, Fast Green and Haemotoxylin

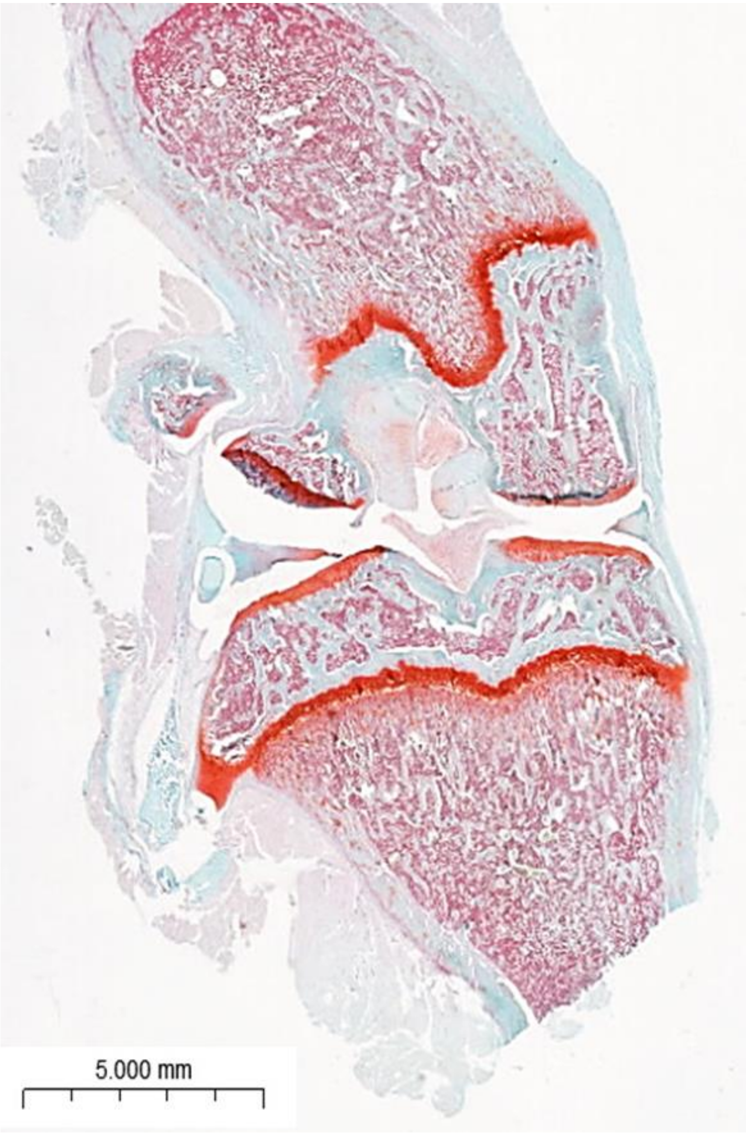

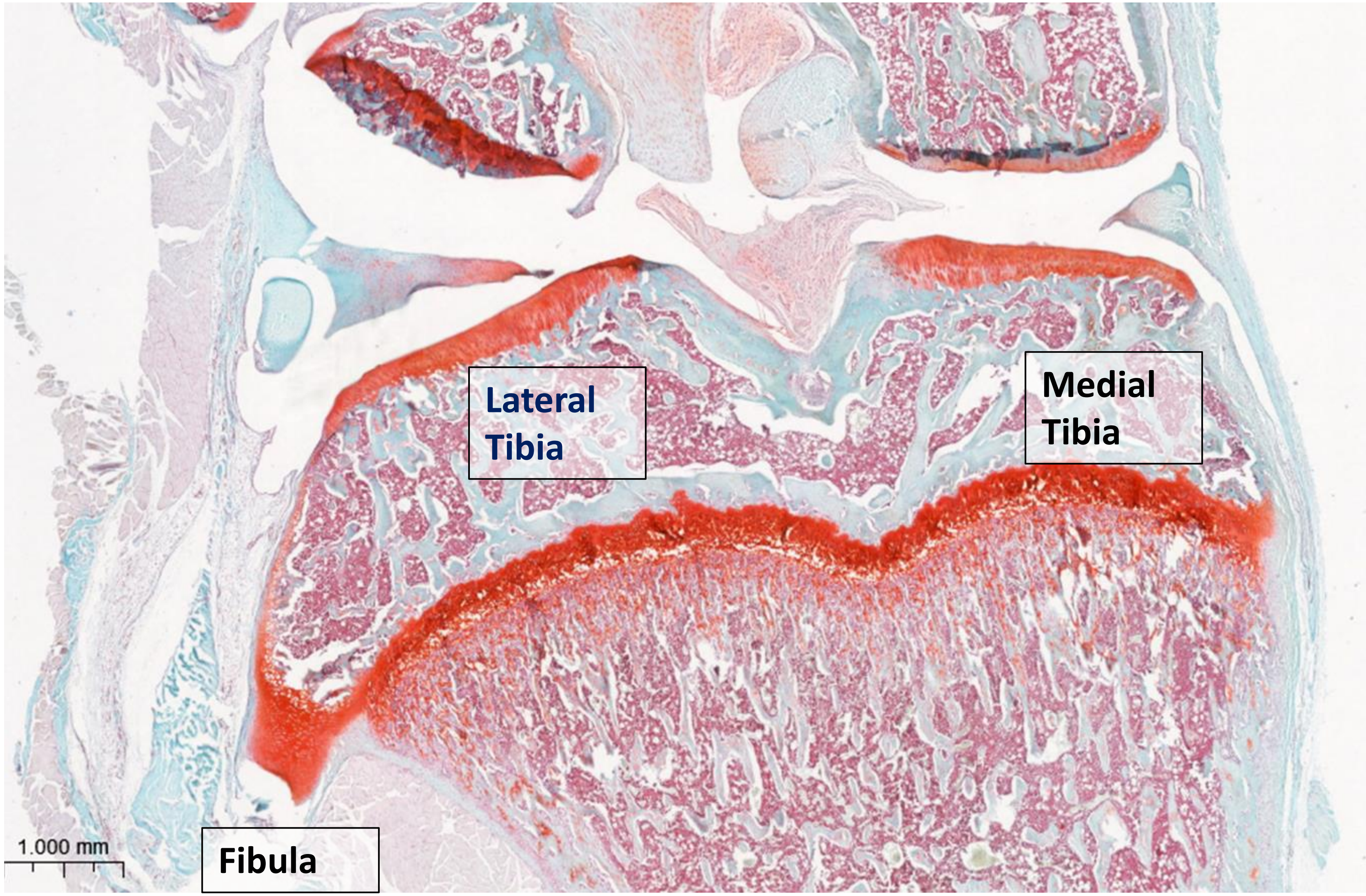

Identifying the region of interest, the subchondral region of the medial tibia

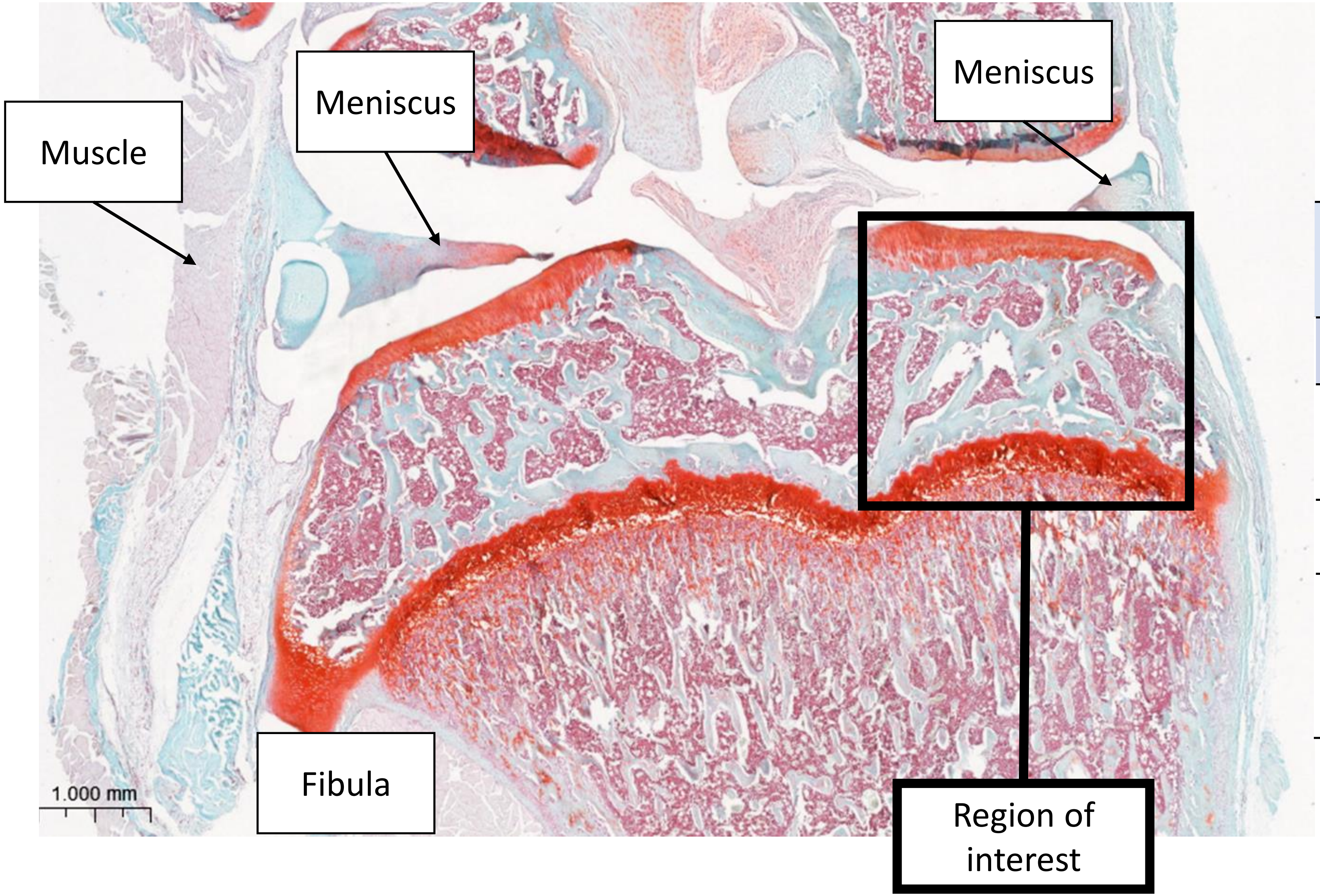

Identification of medial and lateral compartments	
**Lateral**	**Medial**
Fibula	Contralateral to fibula
More muscle	Less muscle
Meniscus has a pointed or angular end	Meniscus might have a pointed or rounded end

Osteoarthritis Bone Score (OABS) in rat knee:- underlying processes
1. Cysts present
2. Fibrosis present
3. Increased blood vessels
4. Cartilage islands present
5. Trabeculae thickened
6. Blood vessels entering non-calcified cartilage
7. Inflammation

Osteoarthritis Bone Score (OABS) in rat knee:- scoring

1. Cysts

None = 0

Present =1

2. Fibrosis

None = 0

Present =1

3. Blood vessels

Normal = 0

Increased numbers =1

4. Cartilage islands

Absent = 0

Present =1

5. Trabeculae thickened

Normal = 0

Increased thickness =1

6. Blood vessels entering non-calcified cartilage

None = 0

At least one blood vessel within articular cartilage =1

7. Inflammation

Normal = 0

Cellular infiltrates present =1

Osteoarthritis Bone Score (OABS) in rat knee subchondral region:- scoring of Present/Absent

1. Cysts
Rounded structures with an outer wall displaying eosinophilic parallel fibres consistent with collagen fibres. Ensure that vessels, adipocytes and sinusoids are not classified as cysts. Scored as present if at least 1 cyst within the subchondral region of interest

2. Fibrosis
Eosinophilic parallel fibres consistent with collagen, with or without fusiform mononucleated cells consistent with being fibroblasts. Scored as Present if any bone marrow space displays fibrosis within the subchondral region of interest.

3. Blood vessels
Annular or tubular structures with cellular (endothelial cell) walls, often have erythrocytes within the lumen and often have smooth muscle around them. Sinusoids are not counted as vessels. Scored as Present if >15 blood vessels within the subchondral region of interest.

4. Cartilage islands.
New cartilage (eosinophilic or safranin-O stained matrix containing chondrocytes) that is discontinuous with articular cartilage. Acellular regions of proteoglycan within bone are not counted as cartilage islands. Scored as Present if at least 1 cartilage island with chondrocytes is within the subchondral region of interest. Do not assess the osteophyte or chondrophyte.

5. Trabeculae thickened.
Scored as present if at least 3 trabeculae show increased thickness (>50 µm at the widest point between junctions with other trabeculae) between the subchondral bone plate and growth plate. Disregard other trabecular abnormalities (e.g. disorganisation).

6. Vessels entering the cartilage
Scored as Present if at least 1 channel (cellular tissue forming a tubular structure that may contain a blood vessel) enters the articular cartilage. Score as 0 if there is no articular cartilage.

7. Inflammation
Non-fusiform mononuclear cells embeded within eosinophilic tissue. Multinucleated cells, consistent with being osteoclasts, and fibroblast-like cells may be present, but should not be the only cell type. Scored as Present if at least 1 area of inflammation within the subchondral region of interest.

Examples of normal rat knees and OA models

Normal appearance (sham-operated)

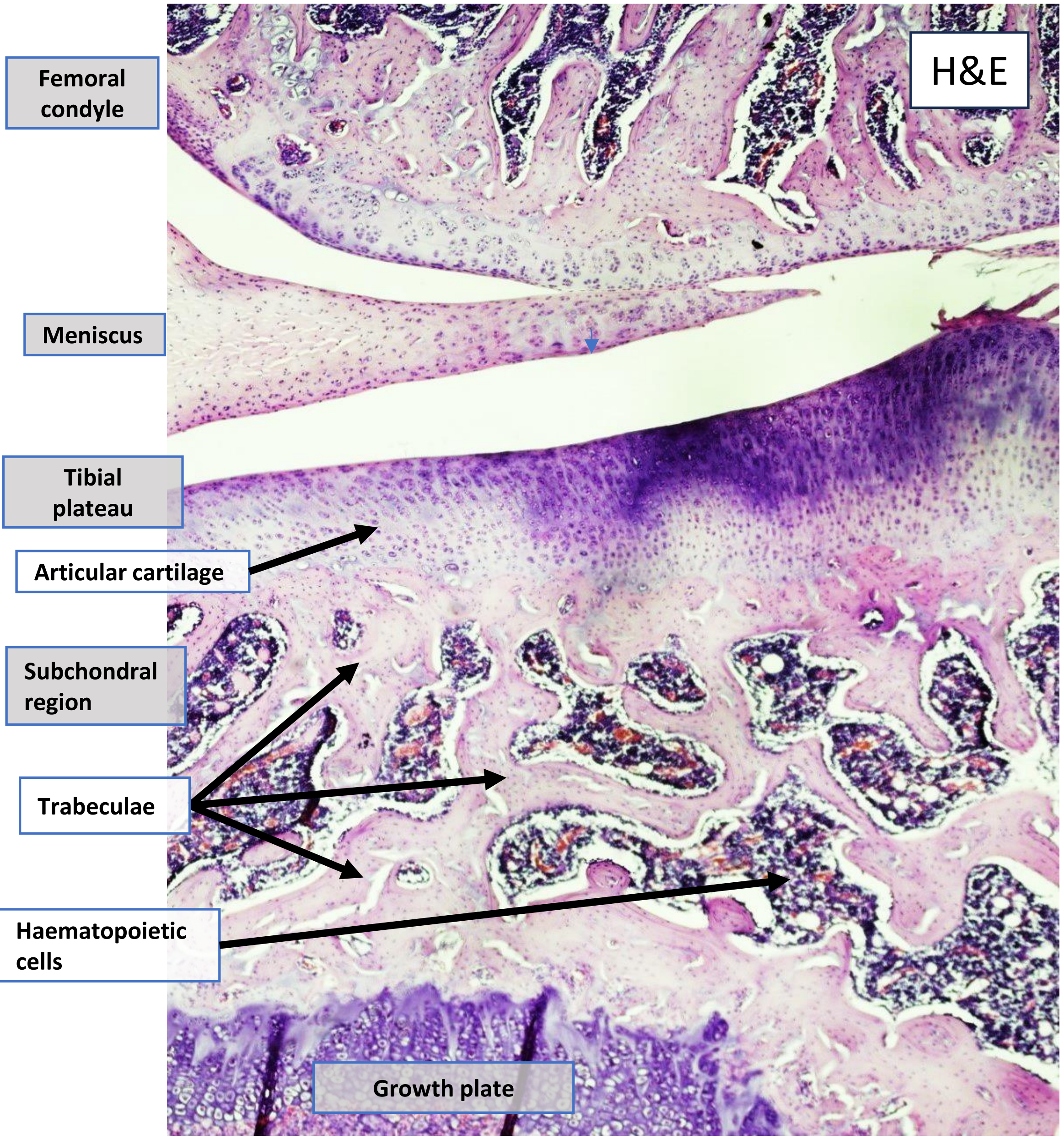

OA model (meniscal transection (MNX))

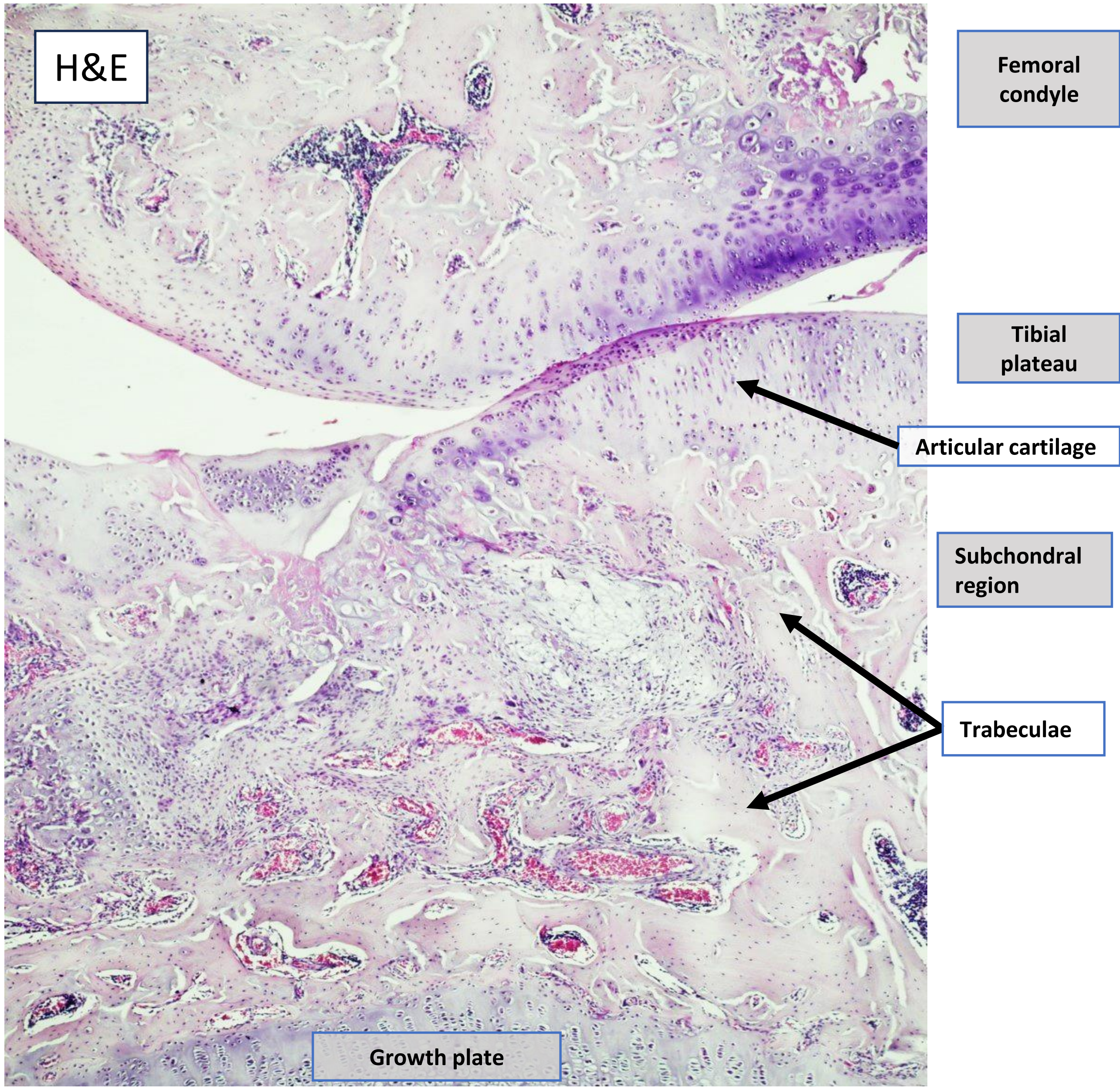

Visual guide for scoring each of the criteria

Cysts present

Score=0

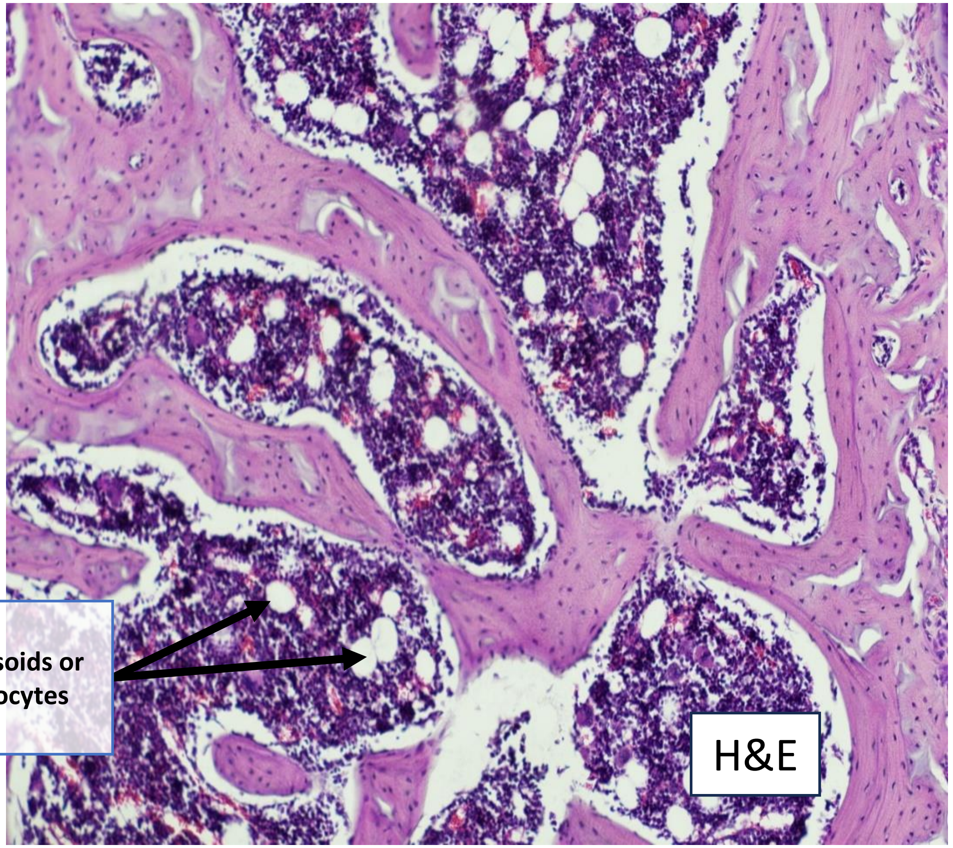

Score= 1

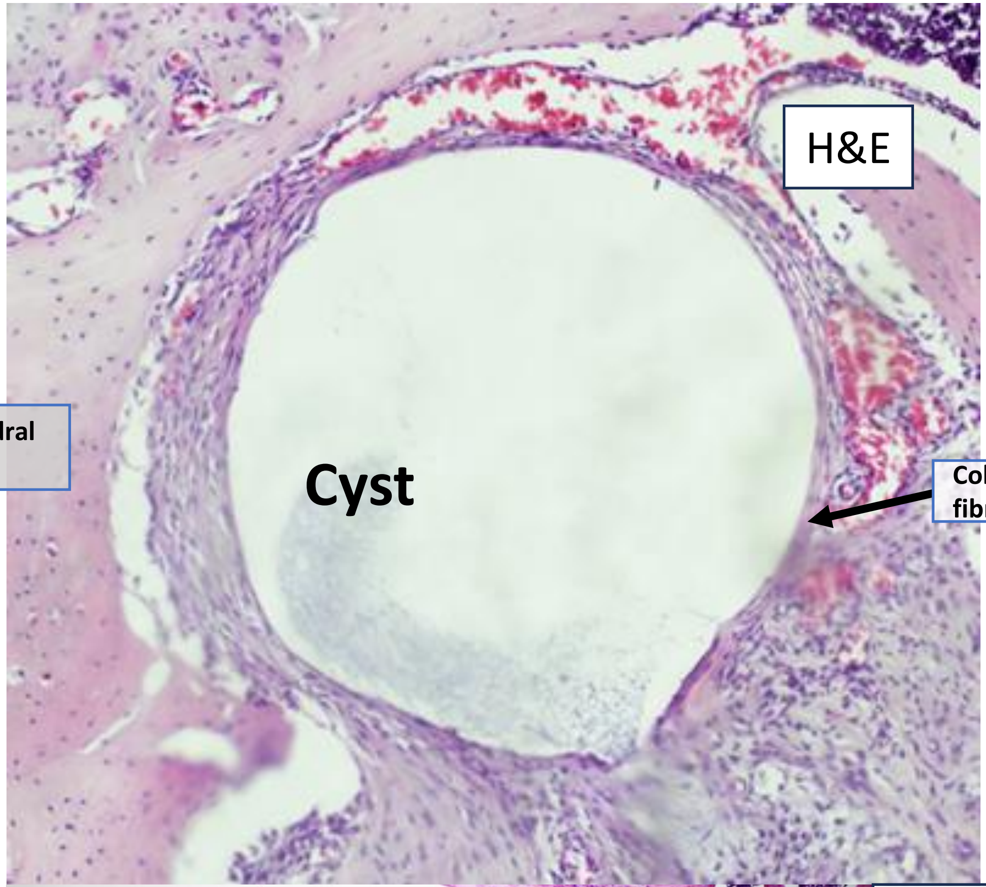

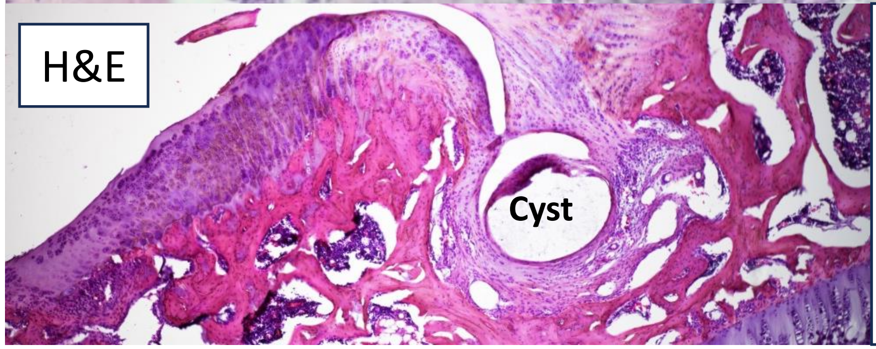

x4 lens objective lens

Cyst at lower and higher magnification

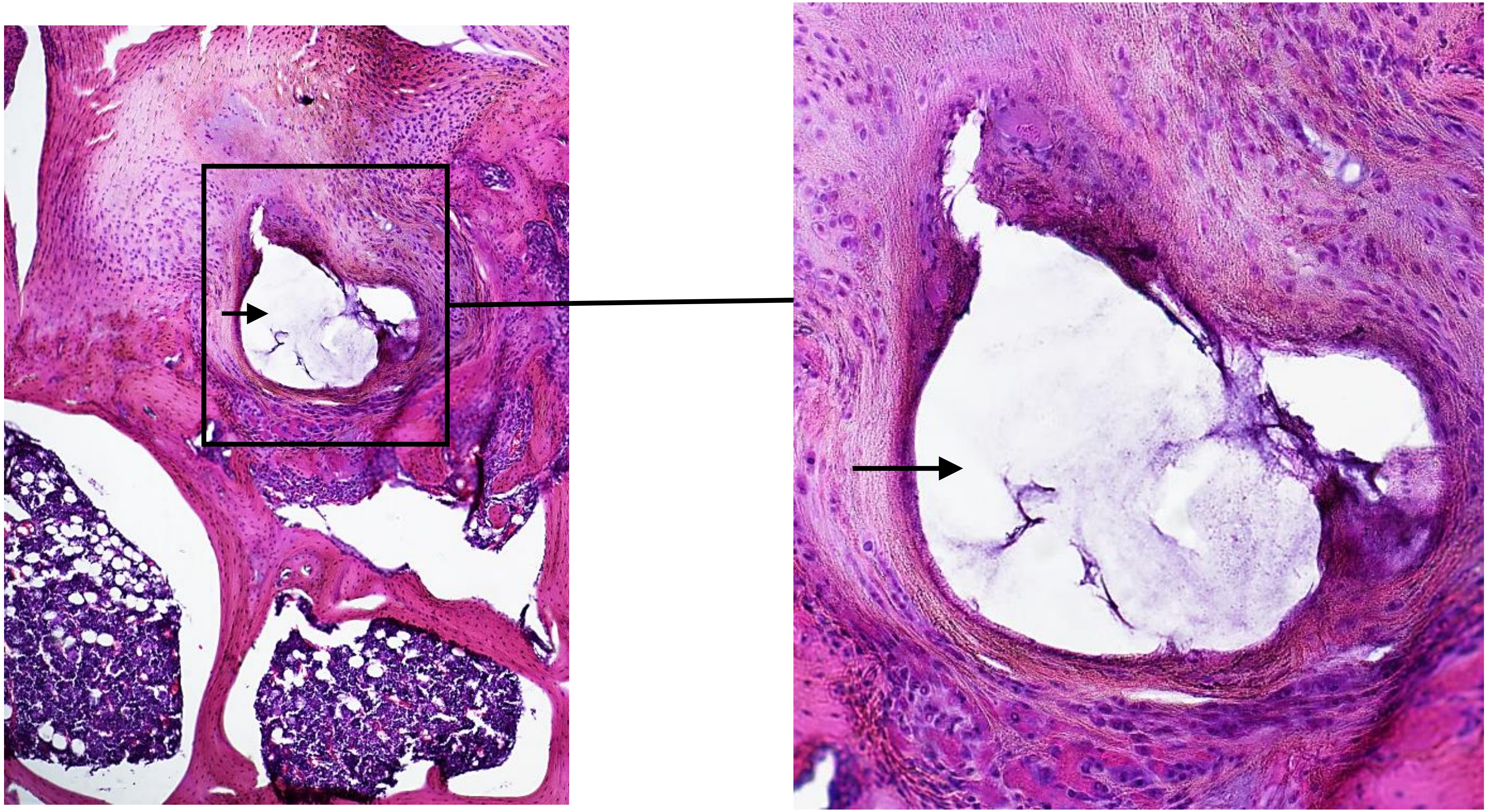

Fibrosis present

Score= 0

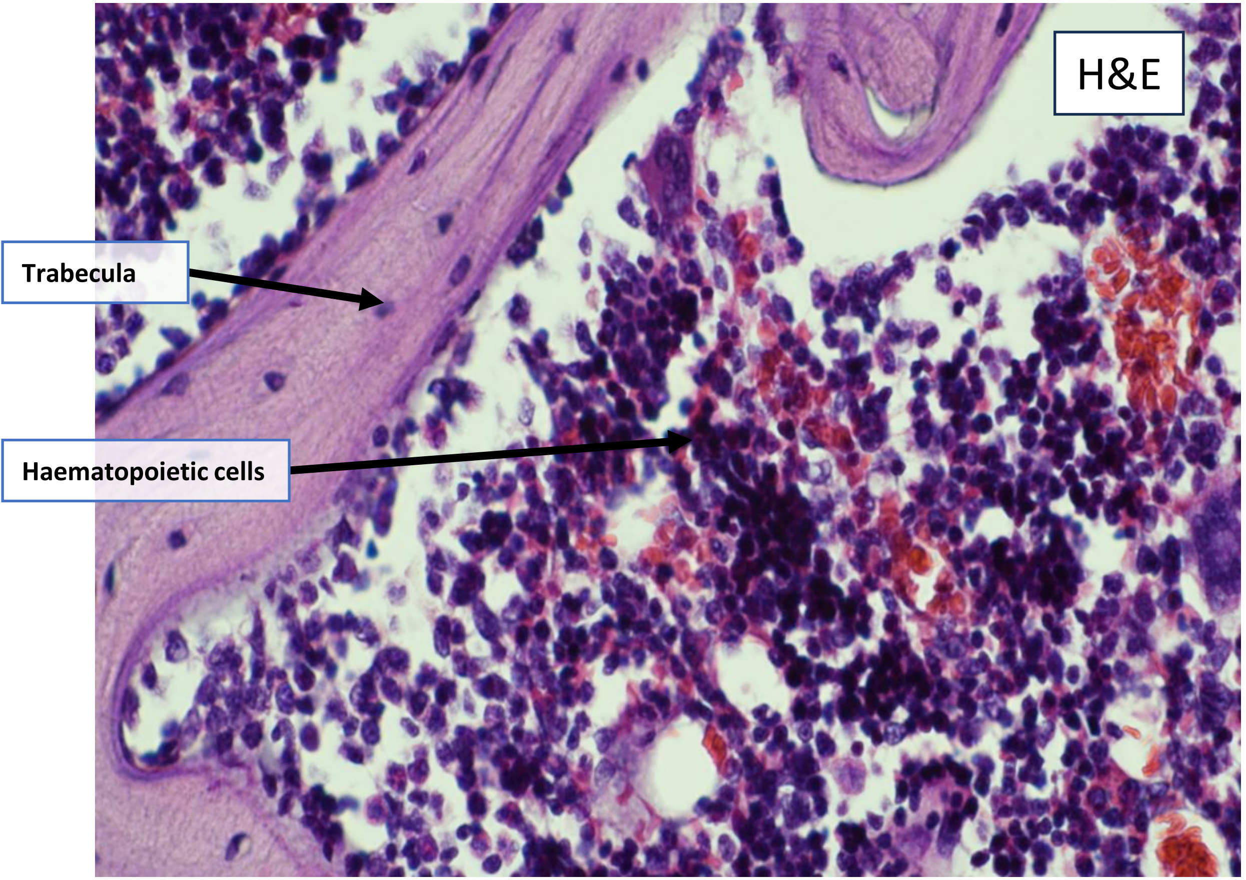

Score= 1

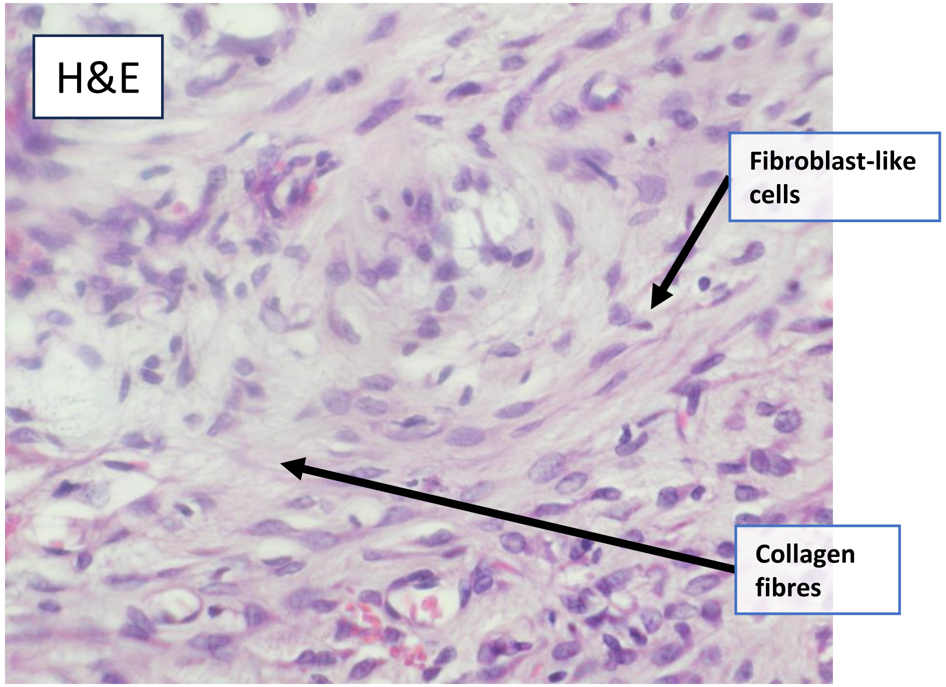

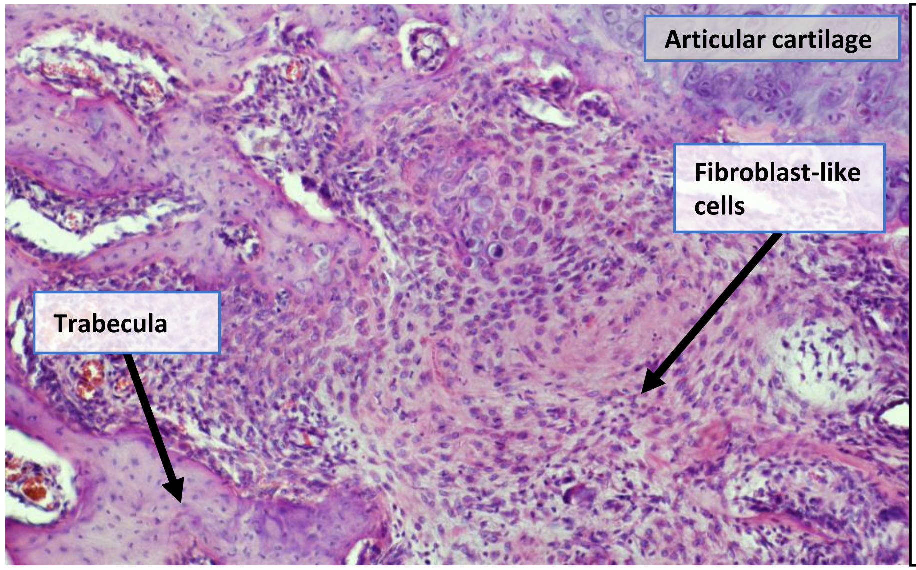

Lower power view of fibrosis

Region of fibrosis at lower and higher magnification

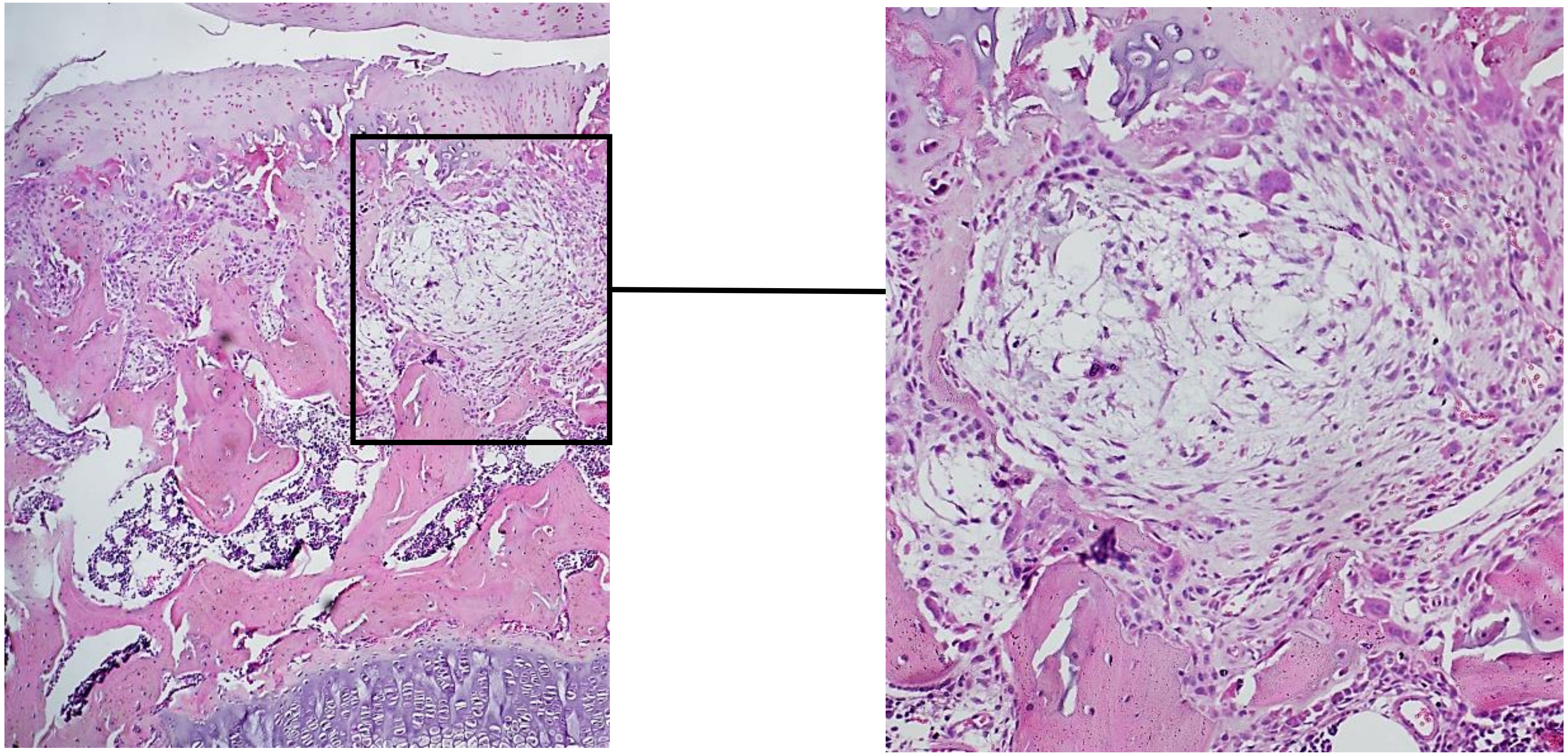

Region of fibrosis and lower and higher magnification

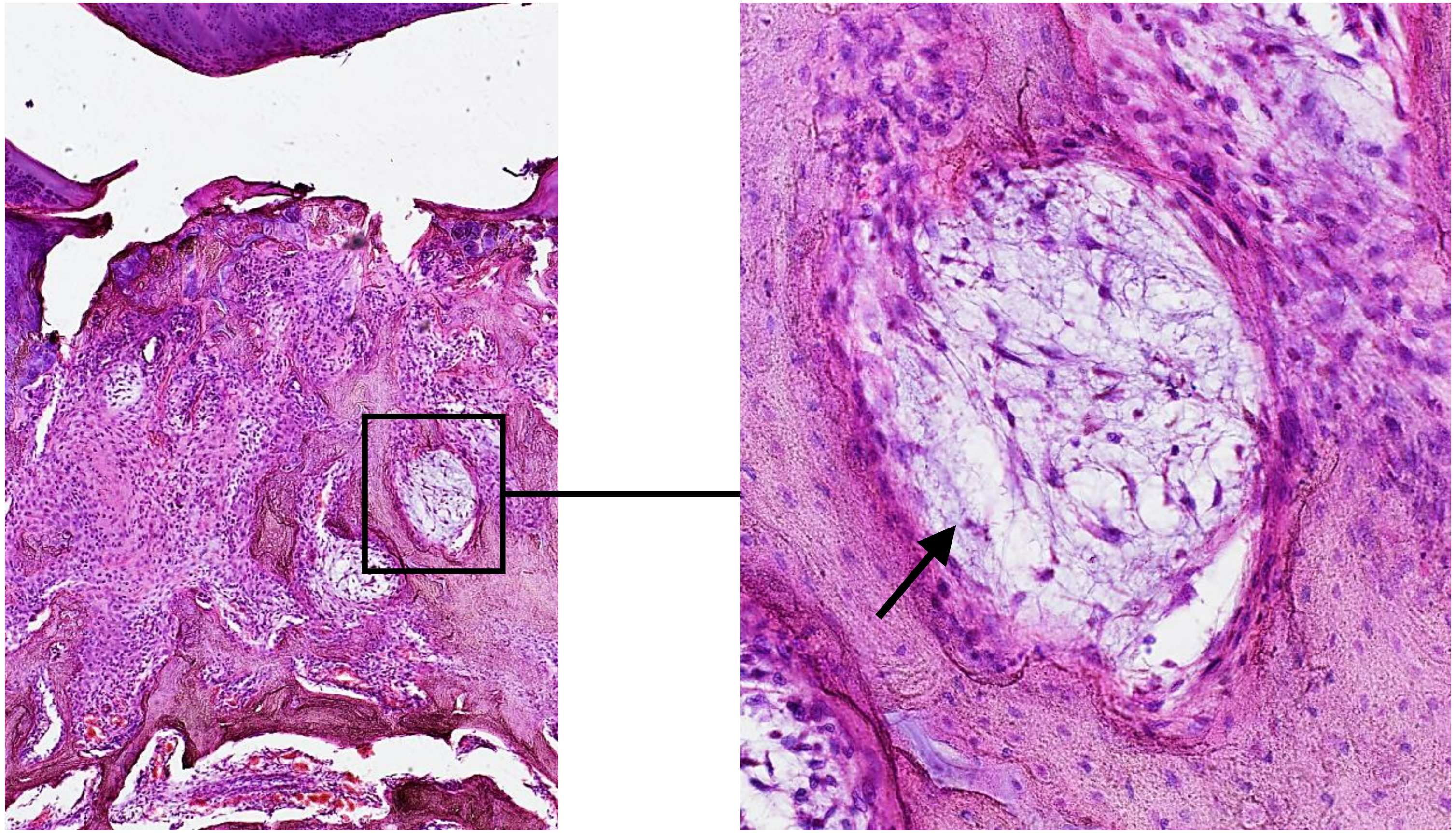

Increased blood vessels

Score= 0

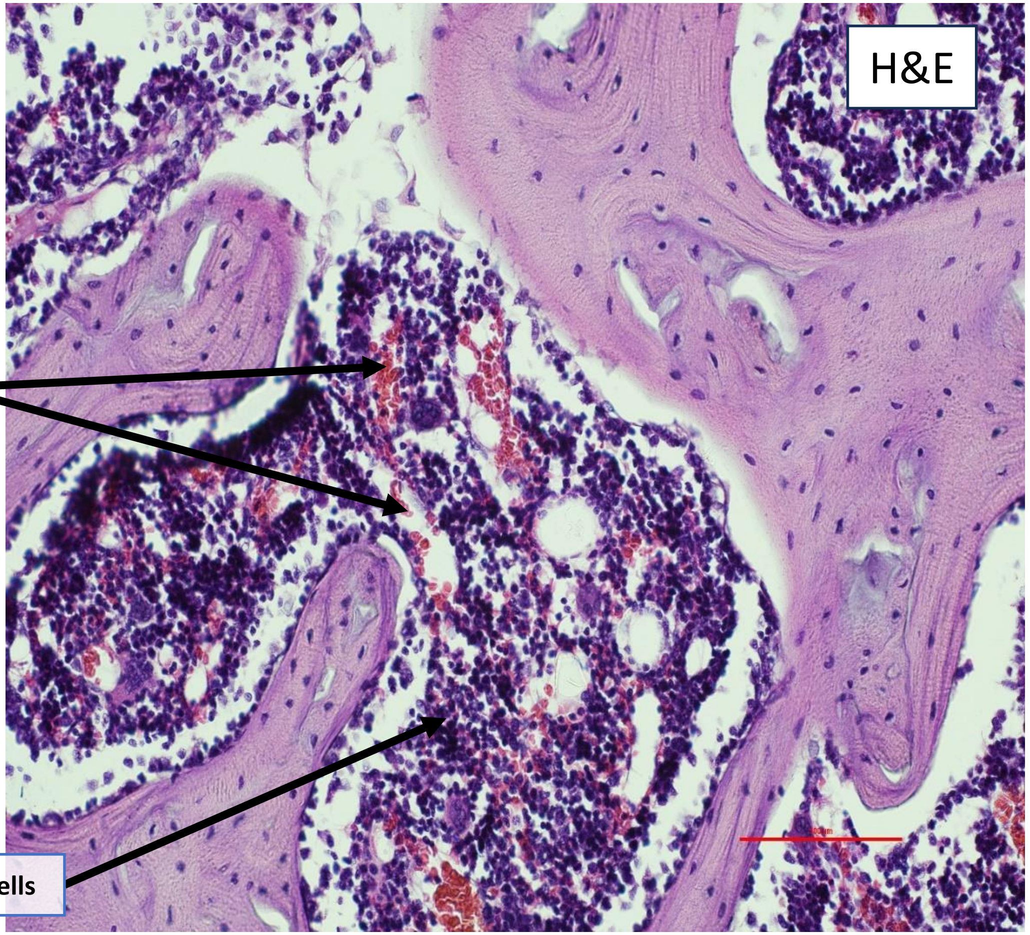

Score= 1

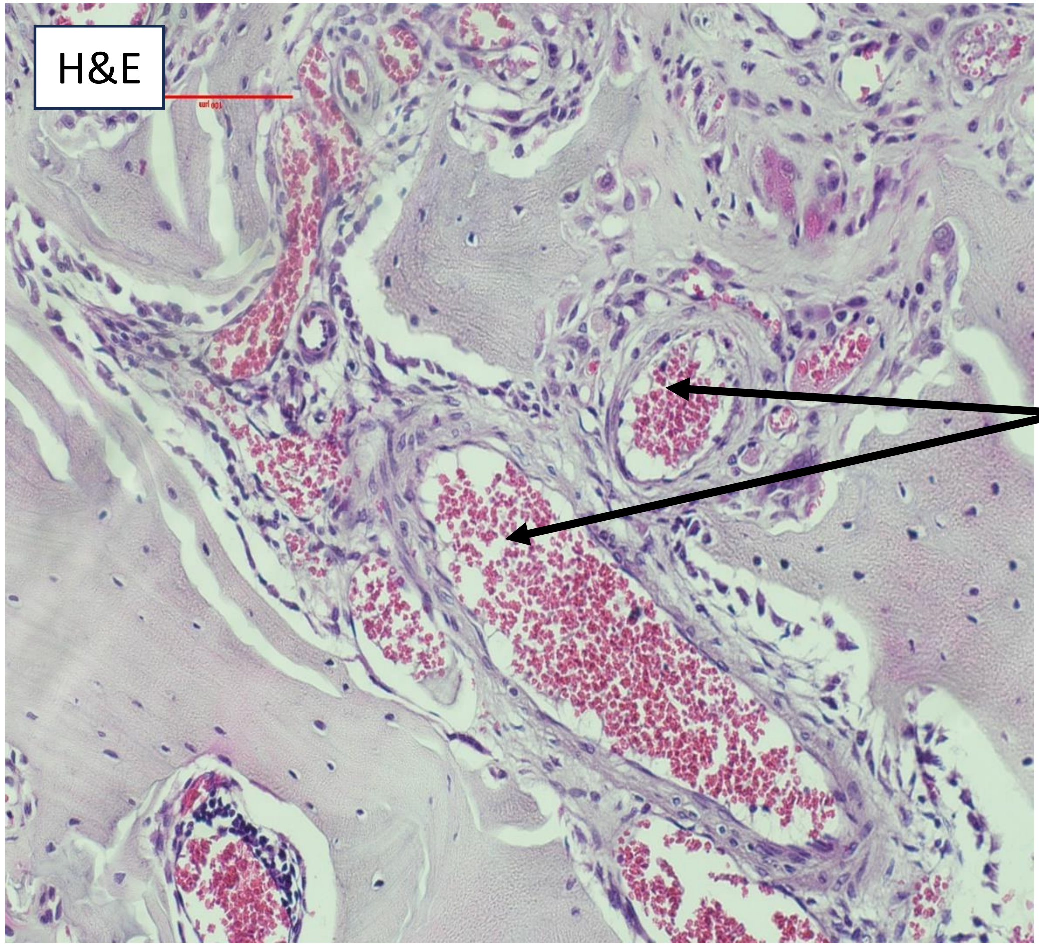

Increased blood vessels viewed at lower magnification

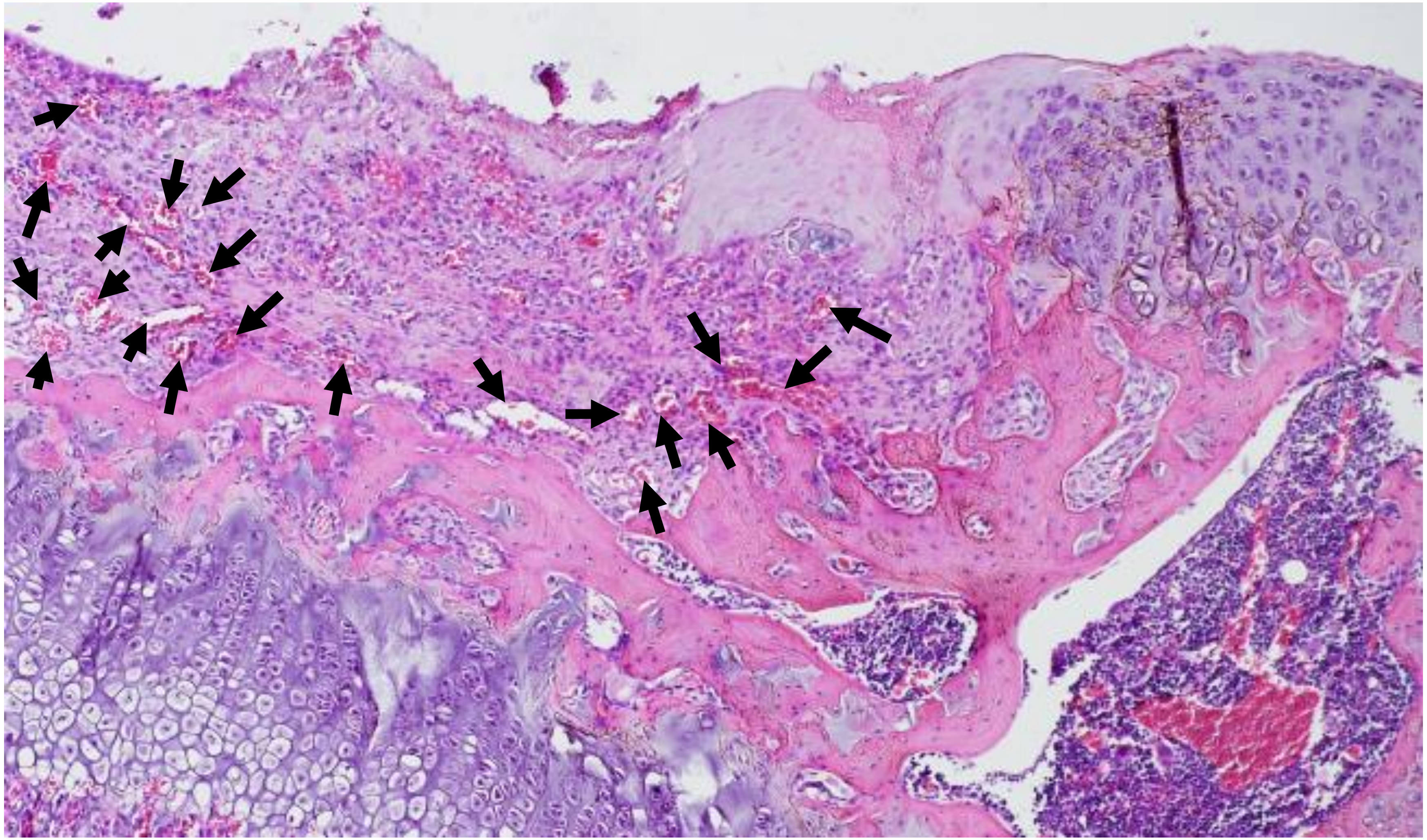

Cartilage Islands present

Score= 0

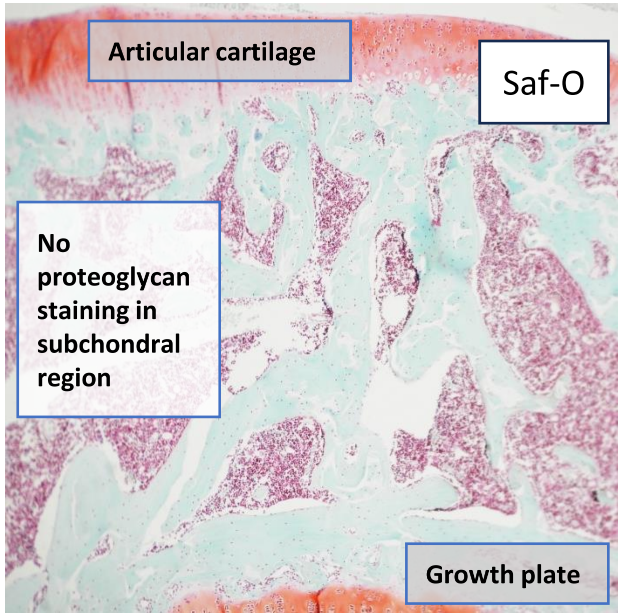

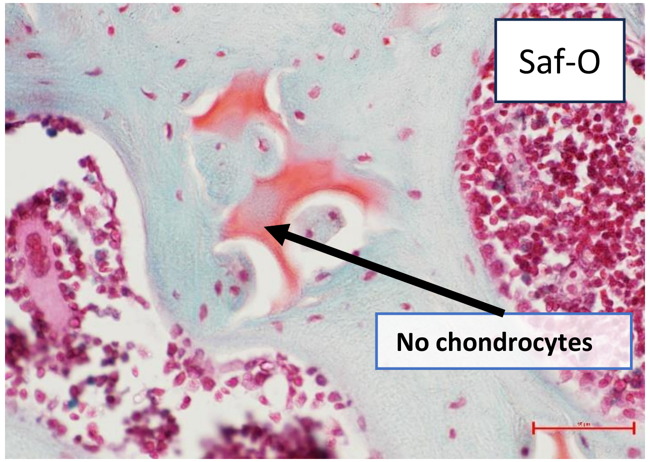

Score= 1

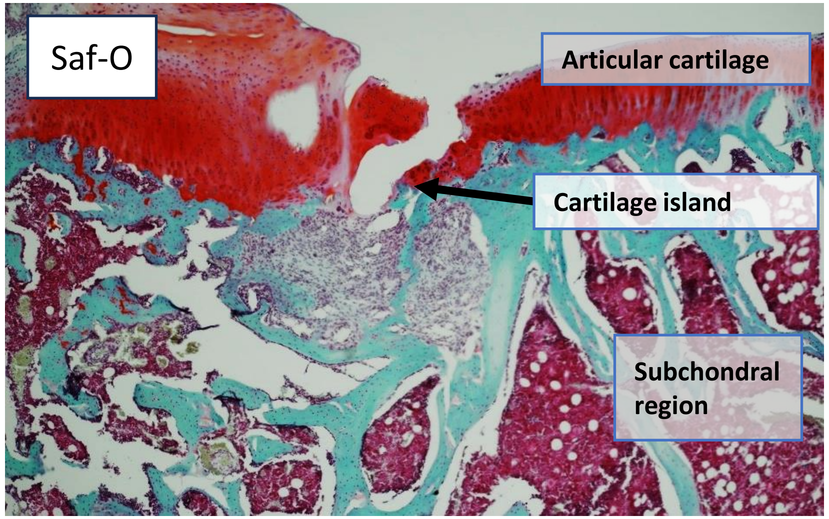

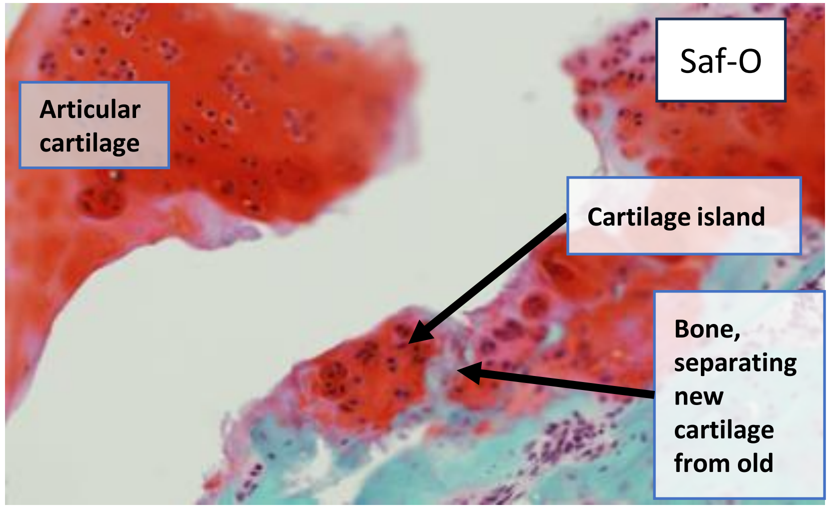

Cartilage islands at lower and high magnification

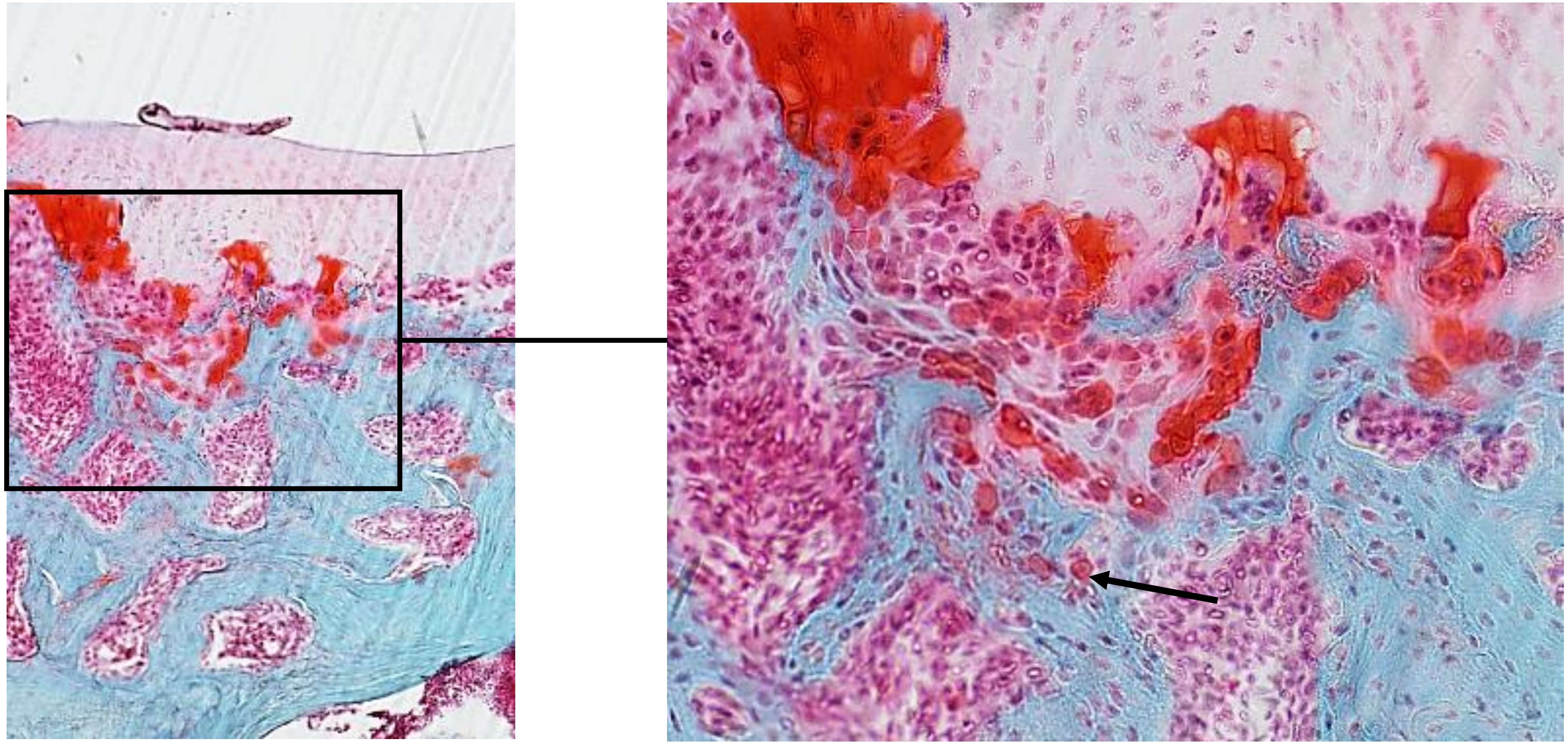

Trabeculae Thickened

Score= 0

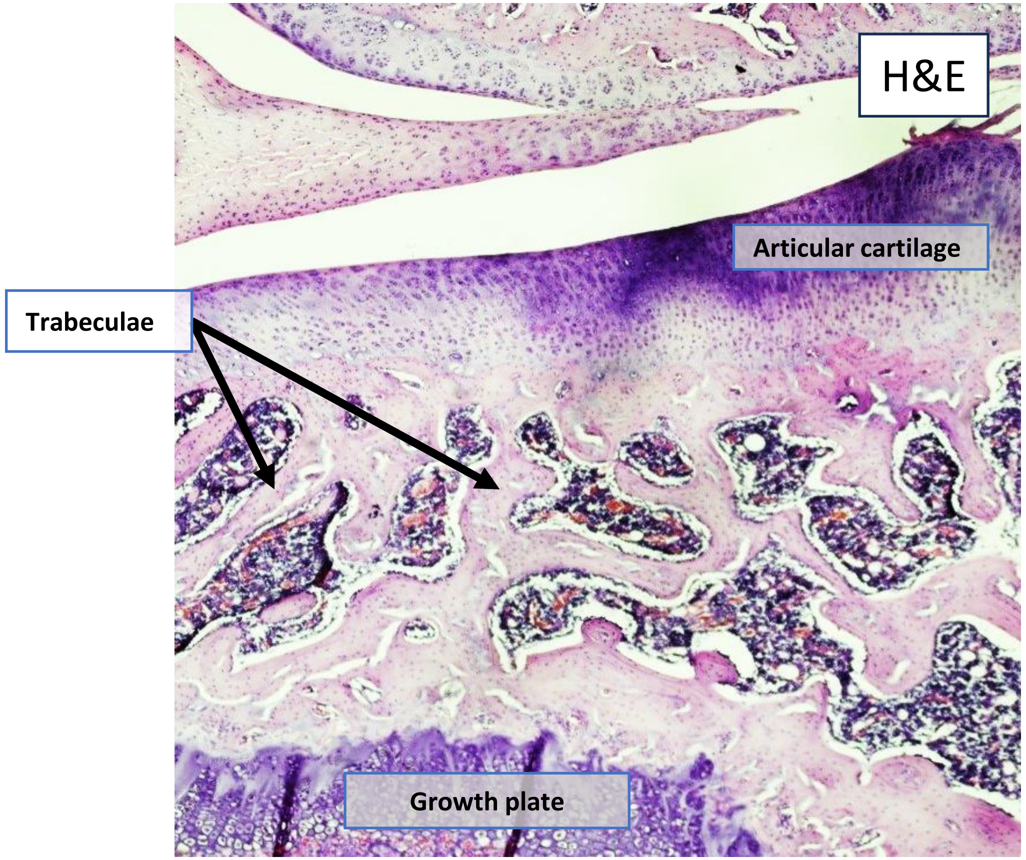

Score= 1

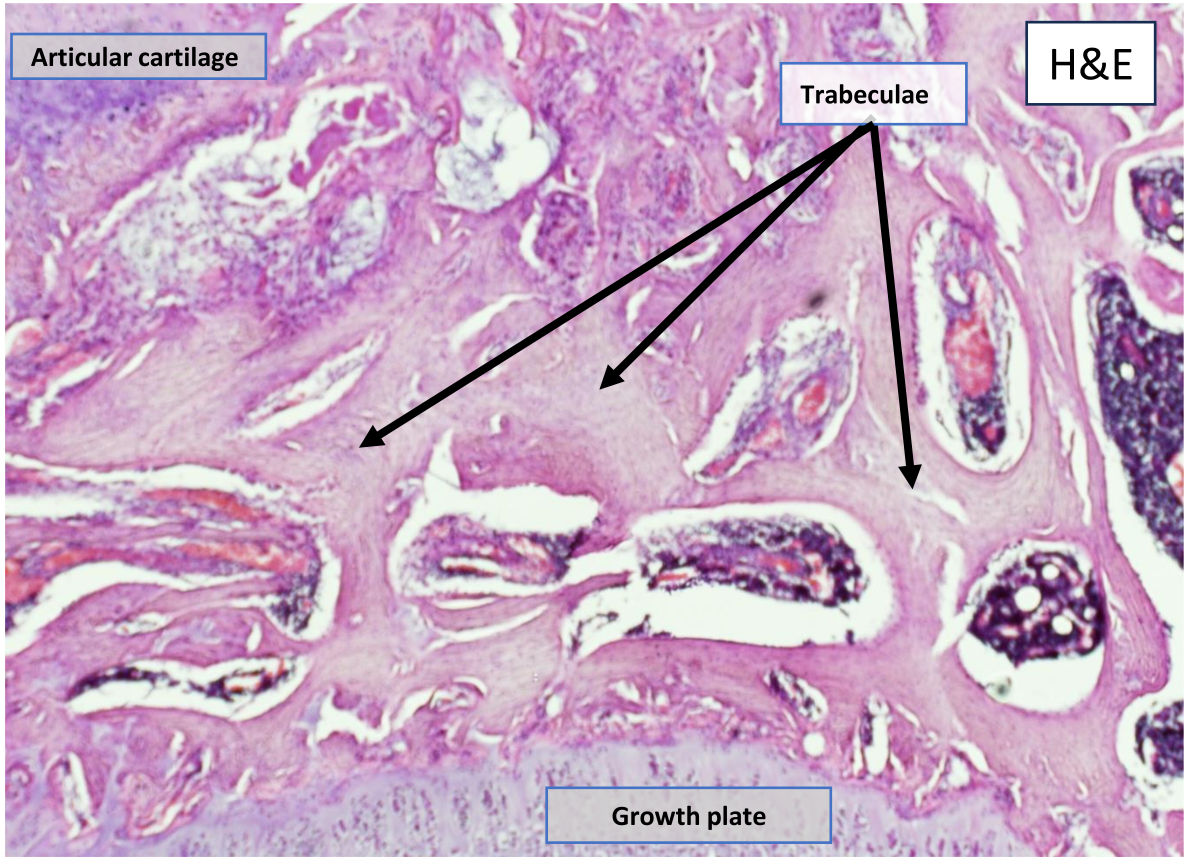

Thickened trabeculae at lower and higher magnification

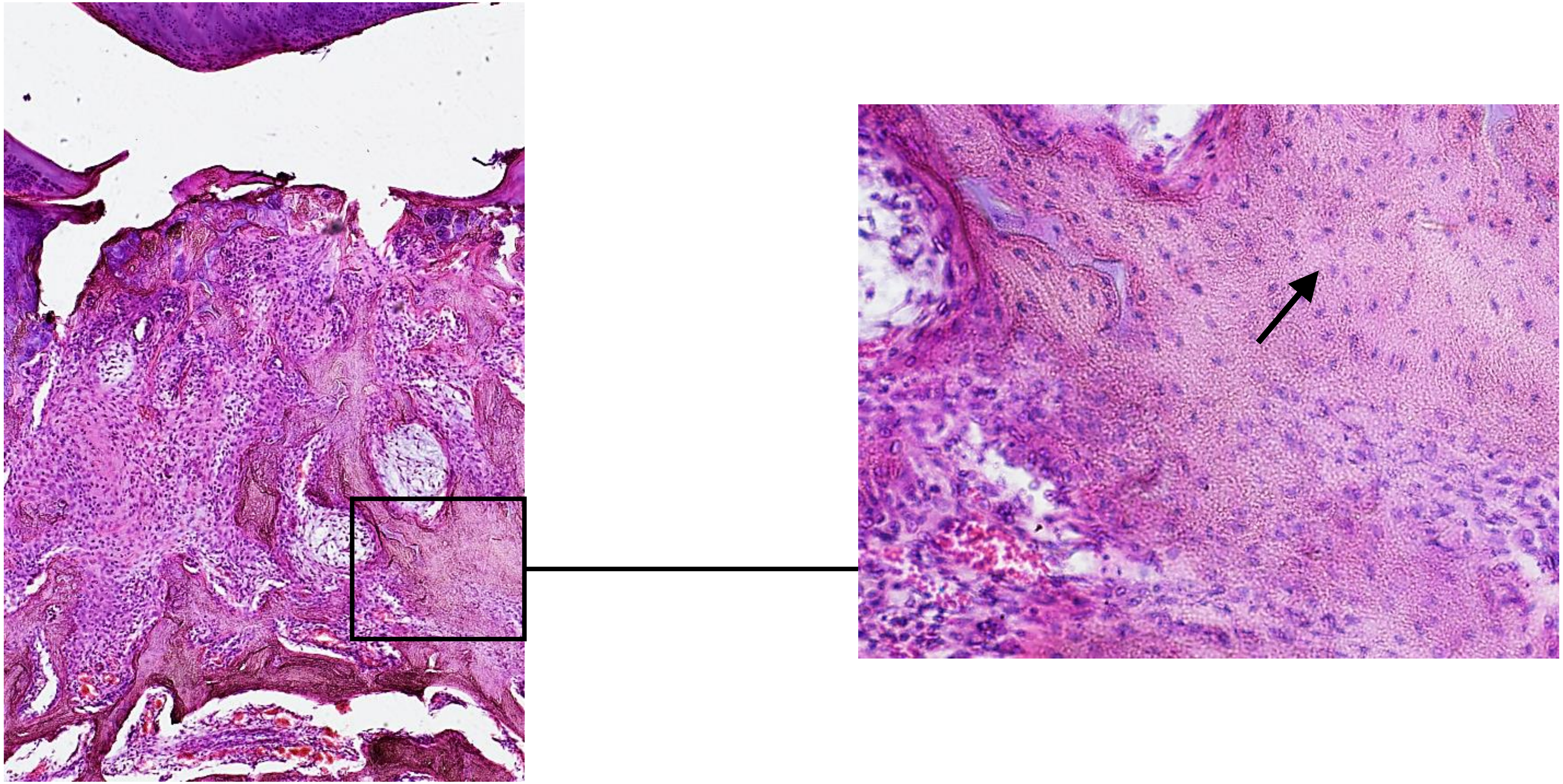

Blood vessels entering non-calcified cartilage

Score= 0

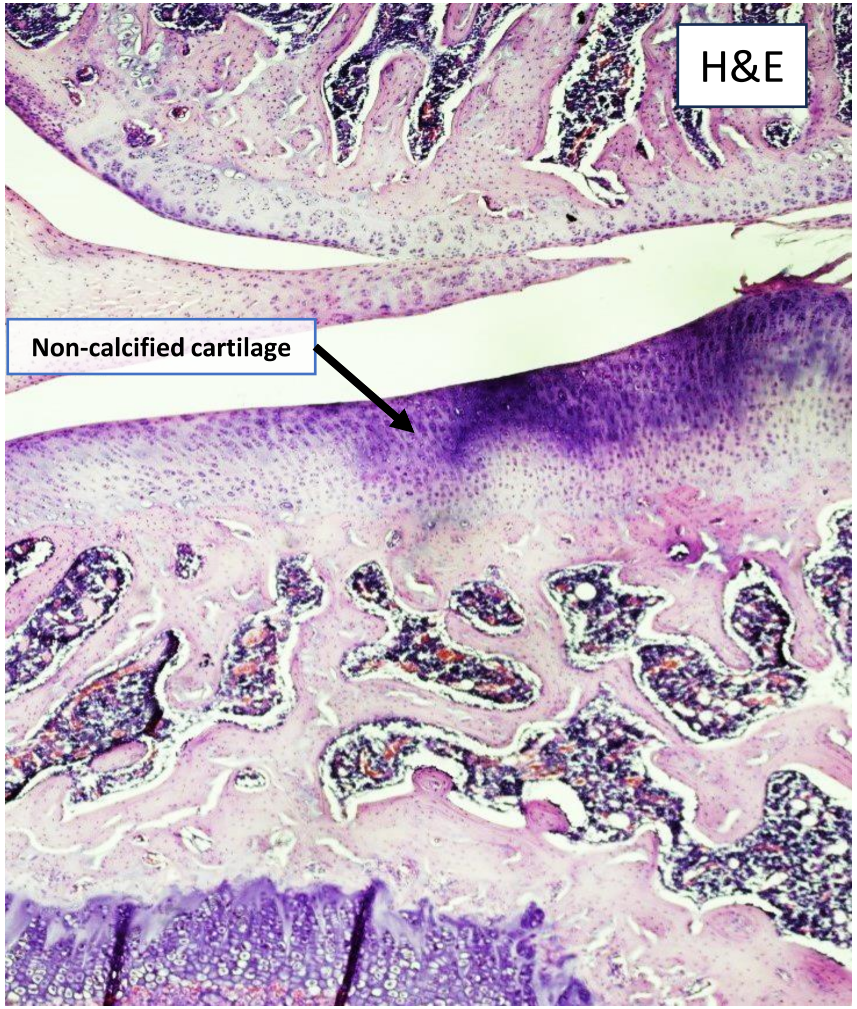

Score= 1

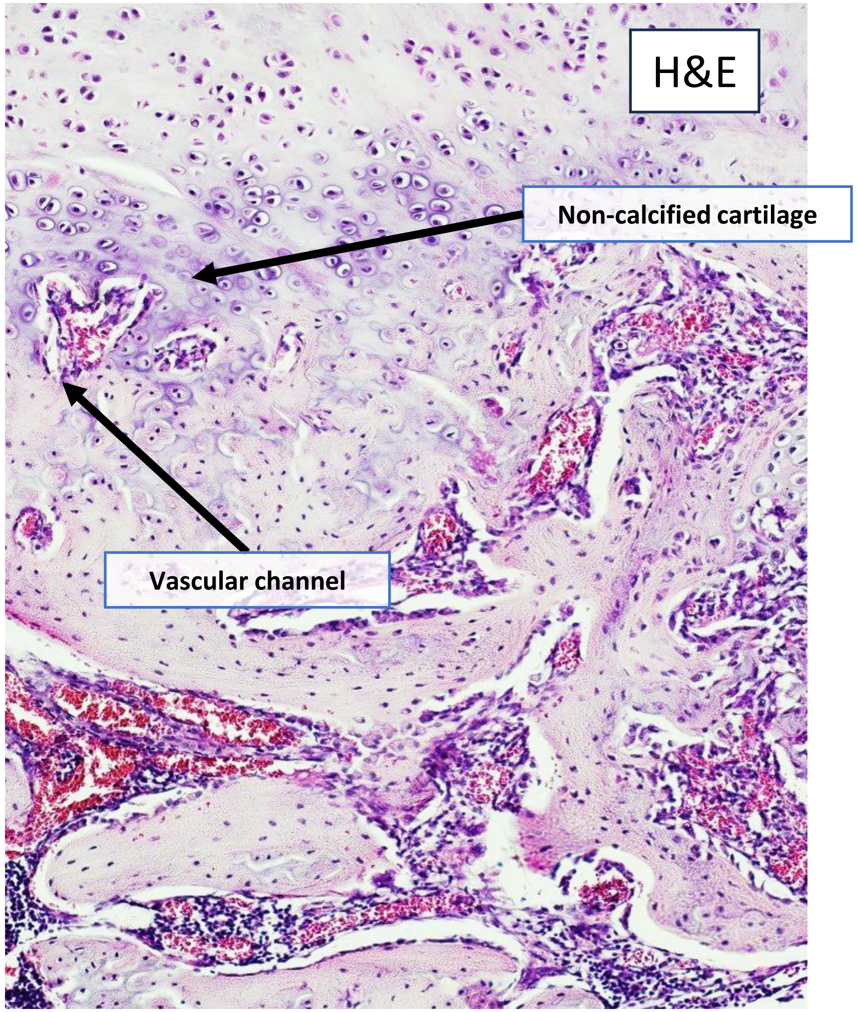

Blood vessel entering non-calcified cartilage at lower and higher magnification

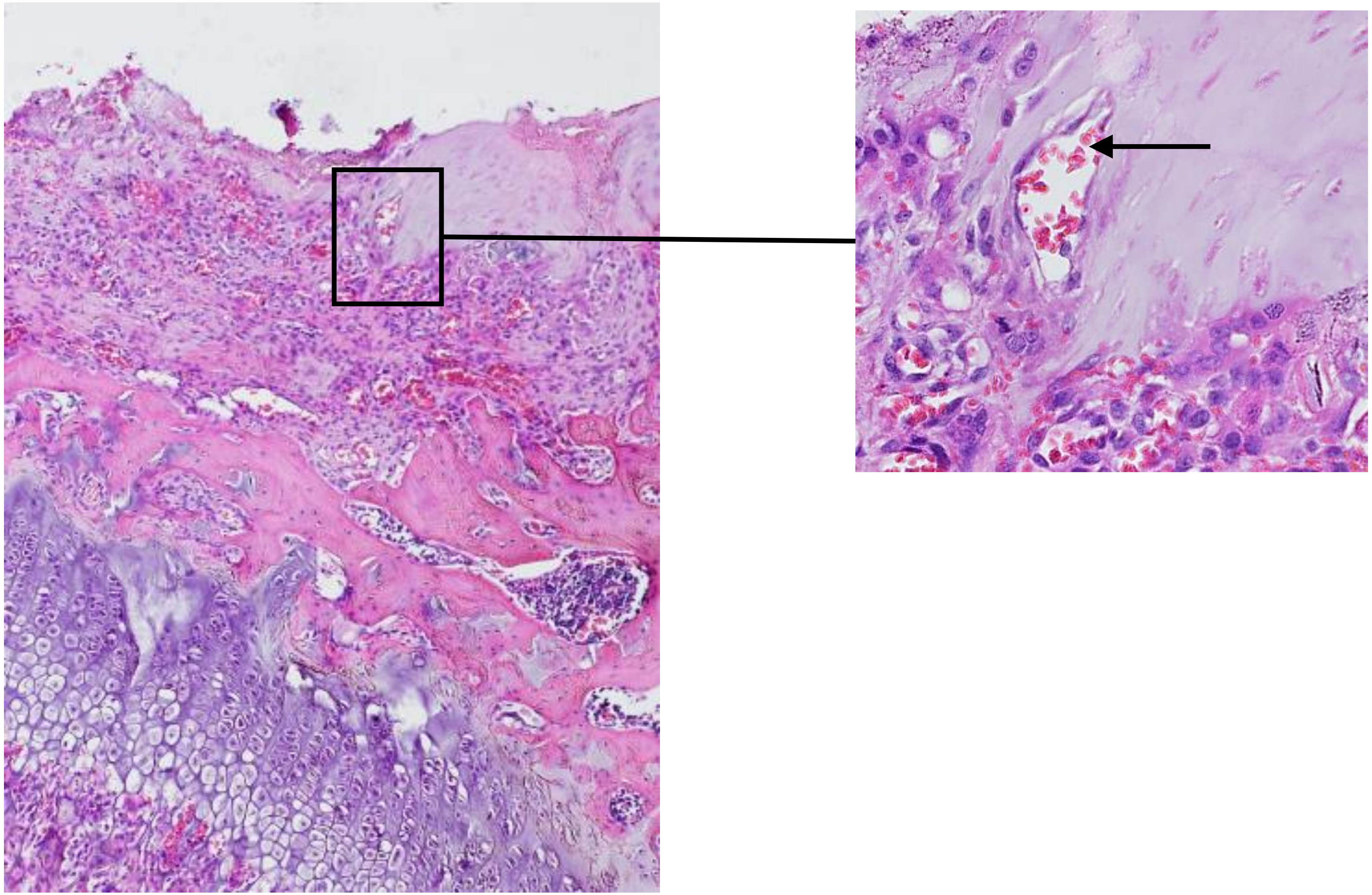

Inflammation

Score= 0

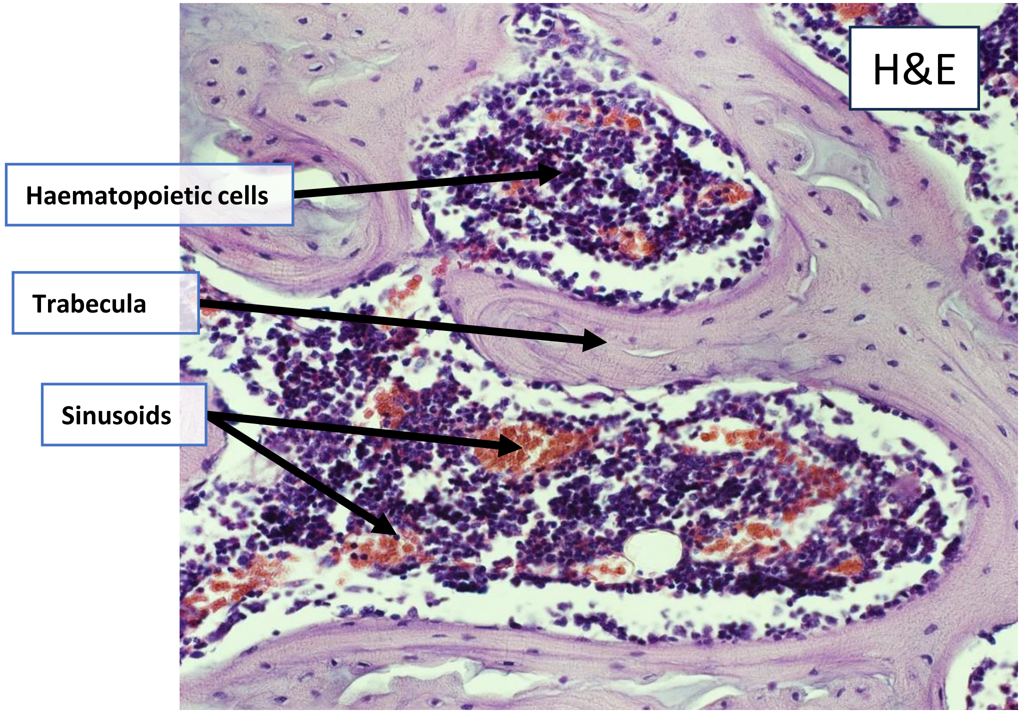

Score= 1

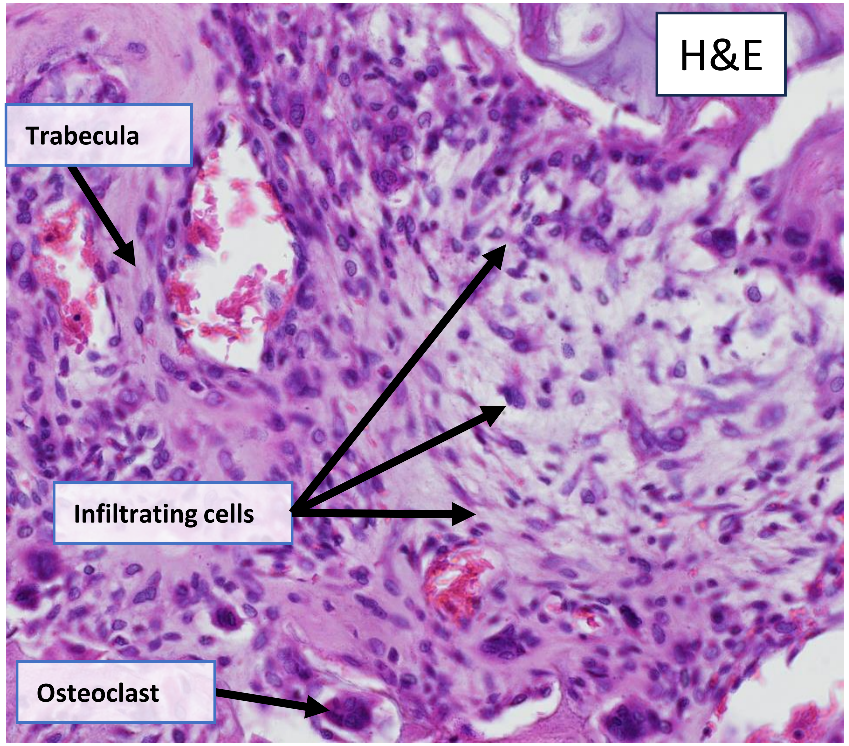

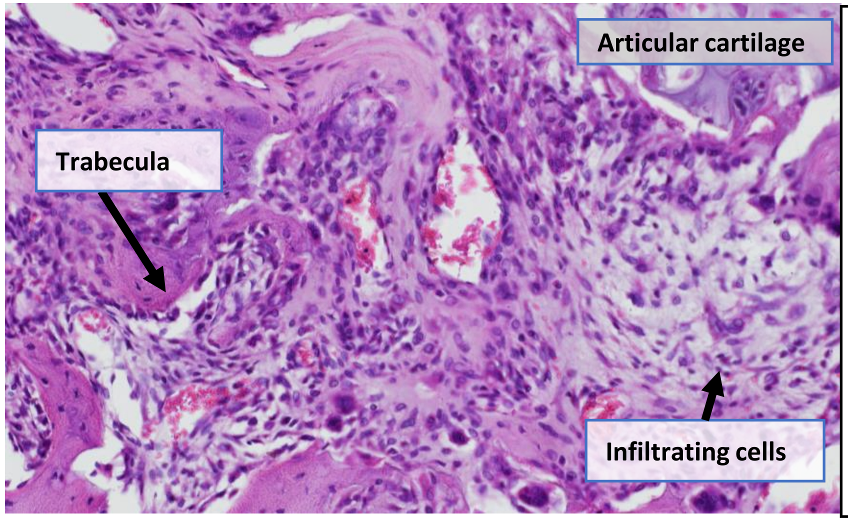

Lower power view

Inflammation at lower and higher magnification

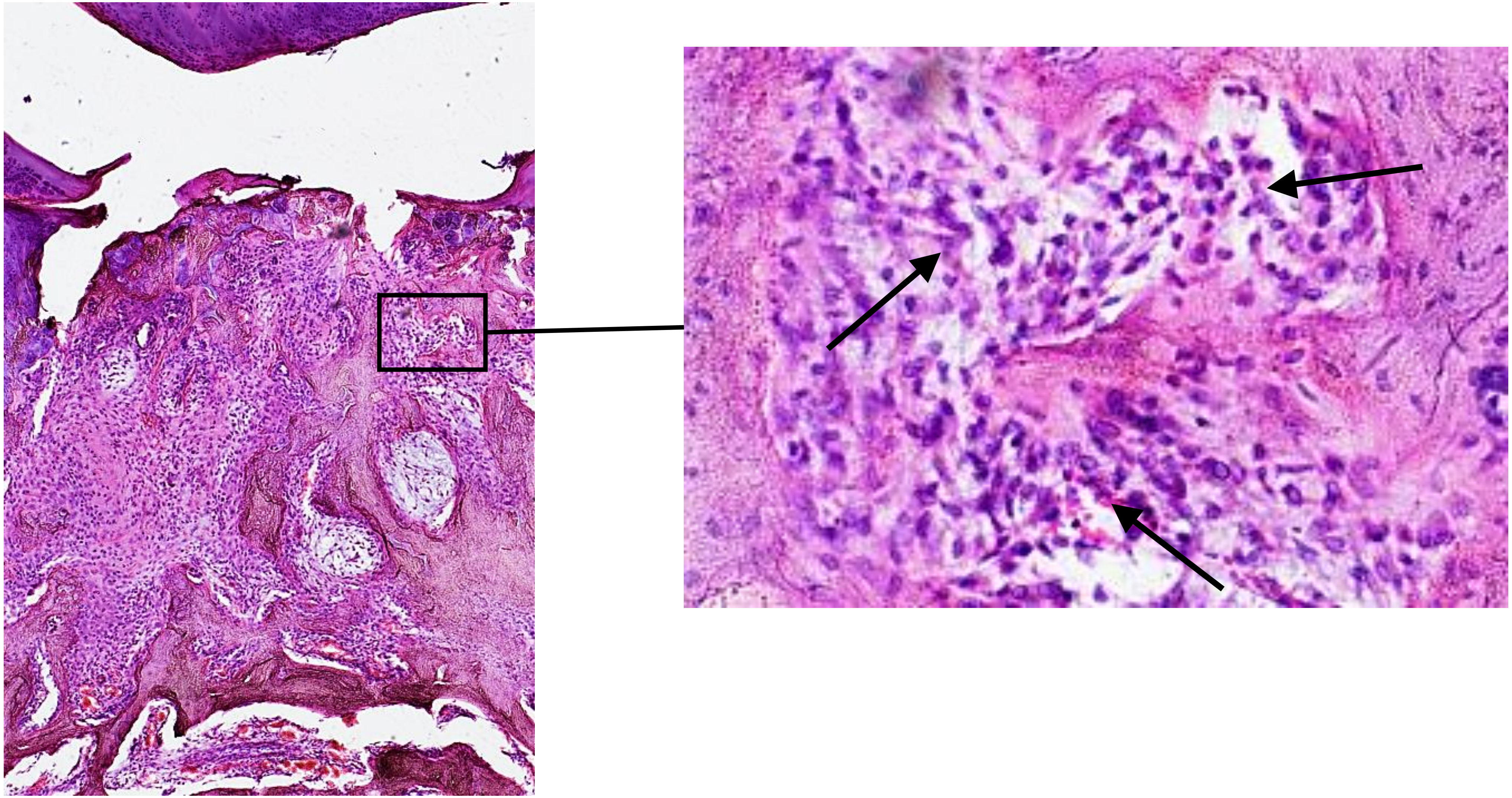

Supplement: Multimedia component 1 [file mmc1.pdf]
