# Supplementary material for: The rat osteoarthritis bone score for histological pathology relevant to human bone marrow lesions and pain
Source: Osteoarthr Cartil Open. 2024 Nov 27;7(1):100544. doi: 10.1016/j.ocarto.2024.100544 (PMC11665527; doi:10.1016/j.ocarto.2024.100544)
Supplement: Multimedia component 2 [file mmc2.docx]

Supplement 2: Mean (95% CI) for differences between groups

|  | **MIA vs vehicle** | **P** |  | **MNX vs Sham surgery** | **p** |  | **MIA vs MNX** | **p** |
| --- | --- | --- | --- | --- | --- | --- | --- | --- |
| **Osteoarthritis features** | | |  |  |  |  |  |  |
| Cartilage  Involvement score | 4.1 (0.4 to 7.8) | 0.033 |  | 4.9 (1.3 to 8.5) | 0.013 |  | 0.2 (-4.5 to 4.9) | 0.930 |
| Cartilage score | 2.0 (0.5 to 3.5) | 0.012 |  | 3.3 (1.2 to 4.4) | 0.003 |  | 0.5 (-1.5 to 2.5) | 0.597 |
| Involvement score | 1.1 (0.3 to 1.9) | 0.013 |  | 1.2 (0.4 to 2.0) | 0.009 |  | 1.1 (0.3 to 1.9) | 0.013 |
| Osteophyte score | Not calculable |  |  | 1.5 (0.5 to 2.5) | 0.007 |  | 1.5 (0.5 to 2.5) | 0.007 |
| Synovial inflammation score | 1.78 (0.7 to 2.7) | 0.004 |  | 2 (1.0 to 3.0) | 0.001 |  | 0.2 (-1.1 to 1.5) | 0.755 |
| **Pain characteristics and measurements** | | |  |  |  |  |  |  |
| Weight bearing asymmetry prior to OA induction (%) | 0.4 (-3.4% to 2.7%) | 0.819 |  | 2.1 (-0.7% to 4.9%) | 0.139 |  | -0.9 (-3.7% to 2.0%) | 0.559 |
| Weight bearing asymmetry during last 7 days prior to termination (%) | 17.5 (-24.3% to -10.6%) | <0.001 |  | -5.8 (-10.2% to -1.4%) | 0.014 |  | 10.7 (3.1 to 18.2) | 0.009 |
| Paw withdrawal threshold prior to OA induction | -3.4 (-10.4 to 3.6) | 0.321 |  | -3.5 (-11.1 to 4.2) | 0.346 |  | -2.9 (-9.4 to 3.6) | 0.378 |
| Paw withdrawal threshold during last 7 days prior to termination (g) | 11.6 (4.9 to 18.3) | 0.002 |  | 1.7 (-4.7 to 8.1) | 0.584 |  | -6.45 (-12.0% to 0.9%) | 0.087 |
| rOABS | 3.1 (1.8 to 4.4) | <0.001 |  | 3.6 (1.9 to 5.3) | 0.001 |  | -0.5 (-2.5 to 1.5) | 0.606 |

Calculated using Welch’s unpaired t-tests with unequal variances assumed. Non-calculable scores due to many duplicate zeros.
